# Organically Templated Uranyl Sulfates and Selenates: Structural Complexity and Crystal Chemical Restrictions for Isotypic Compounds Formation

**DOI:** 10.3390/ijms241613020

**Published:** 2023-08-21

**Authors:** Elizaveta V. Durova, Ivan V. Kuporev, Vladislav V. Gurzhiy

**Affiliations:** Department of Crystallography, Institute of Earth Sciences, St. Petersburg State University, University Emb. 7/9, Saint-Petersburg 199034, Russia; st068755@student.spbu.ru (E.V.D.); st054910@student.spbu.ru (I.V.K.)

**Keywords:** uranyl, sulfate, selenate, isopropylamine, crystal structure, structural complexity, X-ray diffraction

## Abstract

This paper reviews the state of the art in the structural chemistry of organically templated uranyl sulfates and selenates, which are considered as the most representative groups of U-bearing synthetic compounds. In total, there are 194 compounds known for both groups, the crystal structures of which include 84 various organic molecules. Structural studies and topological analysis clearly indicate complex crystal chemical limitations in terms of the isomorphic substitution implementation, since the existence of isotypic phases has to date been confirmed only for 24 compounds out of 194, which is slightly above 12%. The structural architecture of the entire compound depends on the combination of the organic and oxyanion parts, changes in which are sometimes realized even while maintaining the topology of the U-bearing complex. An increase in the size of the hydrocarbon part and number of charge functional groups of the organic cation leads to the formation of rare and more complex topologies. In addition, the crystal structures of two novel uranyl sulfates and one uranyl selenate, templated by isopropylammonium cations, are reported.

## 1. Introduction

Crystal chemical studies of uranium compounds began to develop actively in the middle of the last century; however, the most rapid growth of structural research occurred at the turn of the century and continues to this day. Of particular interest from the structural chemistry point of view is the study of hexavalent uranium compounds. The unique structural diversity cannot leave indifferent researchers in the field of crystallography, thereby generating new discovered substances and new published papers every year. Two of the most abundant groups of synthetic U-bearing compounds are uranyl selenates and sulfates, and a significant portion of them are hybrid organic–inorganic compounds. Their study is of genuine interest, since such complexes inherit the properties of both structural components: a solid inorganic uranium-bearing structure and a flexible organic one.

At present, almost 200 organically templated compounds within both named groups are known (Table 1). In this review, we evaluate the possibility of isostructural compounds’ existence among uranyl sulfates and selenates, as well as involve a recently developed analytical approach to calculating the structural complexity parameters, which allows the comparison of crystal structures in terms of the information content. In addition, we report on a description of the crystal structures of three novel uranyl compounds, [C_3_H_10_N]_2_[(UO_2_)_6_(SO_4_)_7_(H_2_O)_2_] (**1**), [C_3_H_10_N]_2_[(UO_2_)_2_(SO_4_)_3_(H_2_O)](H_2_O) (**3**), and [C_3_H_10_N](H_3_O)[(UO_2_)_2_(SeO_4_)_3_(H_2_O)](H_2_SeO_3_) (**4**), and on the refinement of the previously studied compound [C_3_H_10_N]_2_[(UO_2_)_2_(SeO_4_)_3_(H_2_O)](H_2_O) (**2**) to twice-better convergence parameters and interatomic bonds precision, all of which are templated by isopropylammonium cations, which are reported herein.

## 2. Results and Discussion

### 2.1. Crystal Structure Description

**Figure 1:**
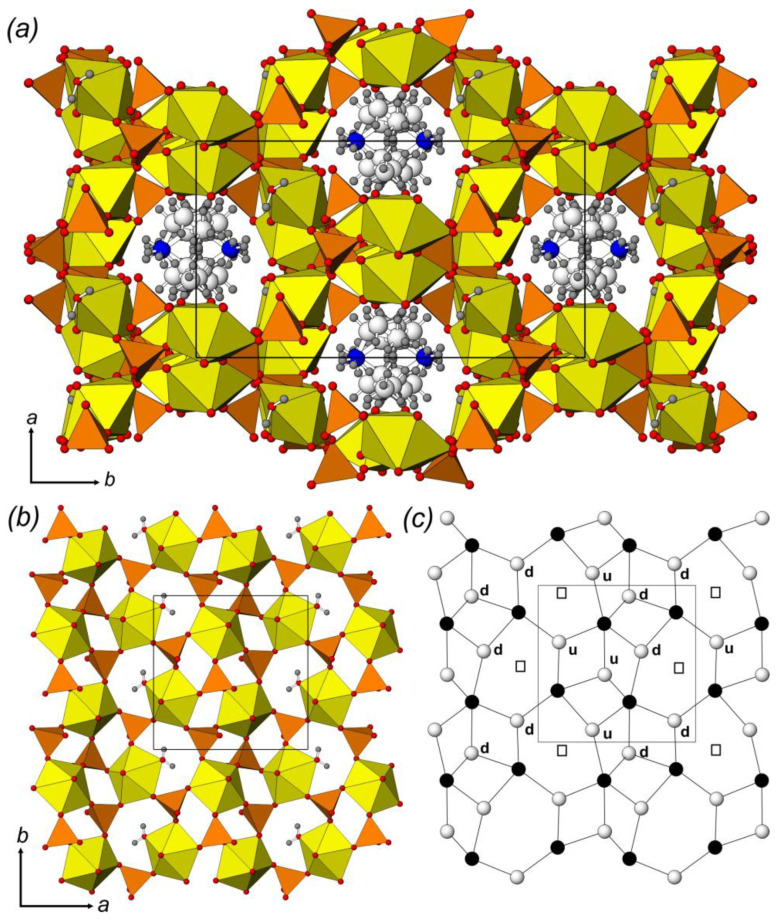
The crystal structure of **1**: (**a**) polyhedral representation of layers in the structures of **2**–**4** (**b**), and topology of its interpolyhedral linkage (**c**). Legend: U polyhedra = yellow, *T*O_4_ (*T* = S, Se) tetrahedra = orange; O atoms = red, N atoms = blue, C atoms = white, H atoms = gray; black nodes = U atoms, white nodes = *T* atoms.

The crystal structure of **1** contains three crystallographically non-equivalent U^6+^ atoms, which are strongly bonded to two O^2−^ atoms, forming linear (within 2.5°) O^2−^≡U^6+^≡O^2−^ uranyl cations (*Ur*) with U^6+^≡O^2−^ bond lengths ranging from 1.738(10) to 1.784(10) Å. The *Ur*1 and *Ur*2 ions are coordinated in the equatorial plane by five O atoms of sulfate tetrahedra, which results in the formation of *Ur*O_5_ pentagonal bipyramids (U1,2–O_eq_ = 2.337(9)–2.449(8) Å). The *Ur*3 ion is coordinated by four O atoms of sulfate tetrahedra and an H_2_O molecule to form a *Ur*3O_4_(H_2_O) pentagonal bipyramid (U3–O_eq_ = 2.337(10)–2.539(9) Å). Four non-equivalent S^6+^ cations are tetrahedrally coordinated by 4 O, each with S^6+^–O^2−^ bond lengths ranging from 1.437(10) to 1.482(9) Å. All sulfate tetrahedra are four-dentate bridging. Uranyl pentagonal bipyramids and sulfate tetrahedra share common edges to form a microporous framework of a [(UO_2_)_6_(SO_4_)_7_(H_2_O)_2_]^2–^ composition (Figure 1a) with elliptical spiral channels passing along the *c*-axis of c.a. 7.6 × 6.8 Å in diameter, if calculated as the shortest distance between terminal O atoms, which is equal to c.a. 4.9 × 4.1 Å of a free diameter (assuming a O^2−^ radii of 1.35 Å). One crystallographically non-equivalent isopropylammonium cation is arranged within the channel, compensating for the negative charge of the framework and forming strong (N–H···O) and weak (C–H···O) H-bonding systems with uranyl and bridging O atoms. The topology of the uranyl sulfate framework in **1** was similar to that found in isotypic uranyl sulfate compounds templated by protonated 1-butylamine [C_4_H_10_N]_2_[(UO_2_)_6_(SO_4_)_7_(H_2_O)_2_] (**28**) [11] and tetramethylammonium [C_4_H_12_N]_2_[(UO_2_)_6_(SO_4_)_7_(H_2_O)_2_] (**74**) [35] cations, as well as in a number of inorganic and organically templated uranyl molybdates [82,83,84].

The crystal structures of **2** and **3** are fully isotypic. There are two non-equivalent U^6+^ atoms, forming *Ur* with U^6+^≡O^2−^ bond lengths falling in the range of 1.757(4)–1.766(3) and 1.763(2)–1.781(2) Å (for **2** and **3**, respectively). The *Ur*1 ions are coordinated in the equatorial plane by five O atoms of selenate/sulfate tetrahedra, which results in the formation of *Ur*O_5_ pentagonal bipyramids (U1–O_eq_ = 2.352(3)–2.438(3) and 2.340(2)–2.440(2) Å, for **2** and **3**). The *Ur*2 ion is coordinated by four O atoms of selenate/sulfate tetrahedra and an H_2_O molecule to form a *Ur*2O_4_(H_2_O) pentagonal bipyramid (U2–O_eq_ = 2.343(3)–2.512(4) and 2.341(2)–2.483(2) Å, for **2** and **3**, respectively). There are three non-equivalent tetrahedral sites occupied by Se^6+^ (**2**) and S^6+^ (**3**) ions that are surrounded by 4 O atoms each with *T*^6+^–O^2−^ bond lengths falling in the range of 1.603(4)–1.653(3) and 1.441(2)–1.496(2) Å (for **2** and **3**, respectively). All tetrahedral groups are three-dentate bridging. Uranyl pentagonal bipyramids and selenate/sulfate tetrahedra share common edges to form a layered complex of [(UO_2_)_2_(*T*O_4_)_3_(H_2_O)]^2–^ (*T* = S, Se) composition (Figure 1b) and are arranged parallel to (001). The negative charge of the layer is compensated by two non-equivalent isopropylammonium cations that are arranged within the interlayer space along with one additional H_2_O molecule.

The crystal structure of **4** is very similar to **2** and **3**. It is also based on the layered complexes of a [(UO_2_)_2_(SeO_4_)_3_(H_2_O)]^2–^ composition with the following bond-length ranges: U≡O_Ur_ = 1.759(4)–1.767(3) Å; U1–O_eq_ = 2.374(3)–2.443(3) Å; U2–O_eq_ = 2.359(3)–2.480(4) Å; and Se^6+^–O = 1.612(4)–1.658(3) Å. The difference between structures **2** and **4** lies in the interlayer space. If there are two isopropylammonium cations and one H_2_O molecule in the structure of **2**, the structure of **4** contains one isopropylammonium cation, one hydronium ion, and an additional selenous acid molecule [H_2_SeO_3_]^0^ with Se^4+^–O = 1.681(4)–1.732(5) Å. It is also of interest that quite unusual interatomic interactions are observed in the structure of **4** between the Se4(IV) atom of the [H_2_SeO_3_]^0^ molecule and O2 of the *Ur*2 ion (Se4···O6 = 3.000(4) Å and O20–Se4···O2 = 172.2(2)°), terminal non-shared O17 atom of the [Se1O_4_]^2–^ selenate tetrahedra (Se4···O17 = 3.112(4) Å and O19–Se4···O17 = 140.4(2)°), O13 of the *Ur*1 ion (Se4···O13 = 3.359(4) Å and O19–Se4···O13 = 148.1(2) °); however, the closest contact is observed between Se4 and O6 of the *Ur*1 ion (Se4···O6 = 2.730(4) Å and O21–Se4···O6 = 176.8(2)°). All these interatomic distances, especially the latter, are lower than the sum of the Se and O van der Waals radii (1.9 + 1.55 = 3.45 Å [85]); therefore, they can be attributed to chalcogen bonding [86,87,88,89].

### 2.2. Structural Topology

**Figure 2:**
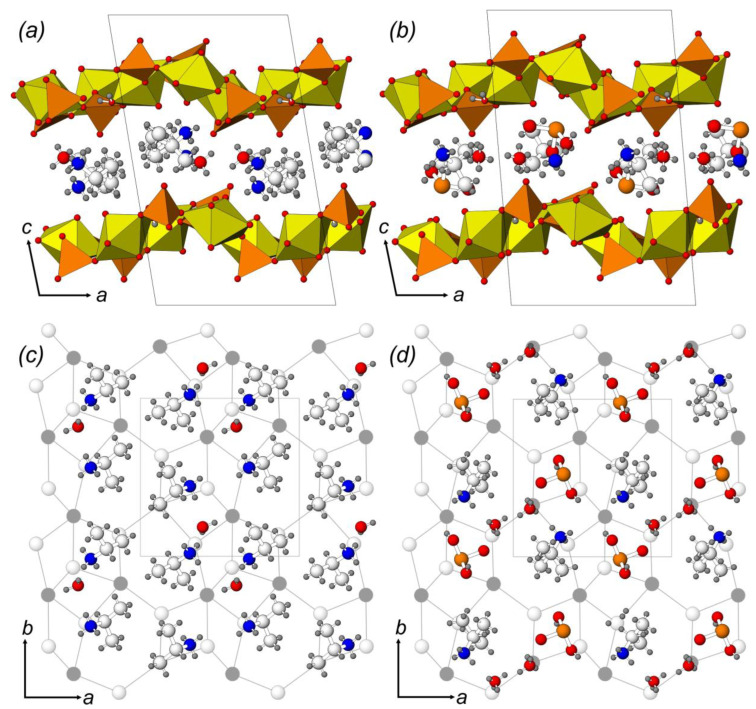
The crystal structures of **2** (**a**) and **4** (**b**): location of the interlayer species in the structures of **2** (**c**) and **4** (**d**) relative to the black-and-white graph of the inorganic layer. Legend: see Figure 1.

The layered complexes in the structures of **2**–**4** belong to one of the most common topological types (*cc*2–2:3–4) among uranyl compounds of both pure inorganic or organically templated origin. The topology of the layer can be represented by a black-and-white graph where *Ur* polyhedra are denoted by black vertices, SO_4_ or SeO_4_ coordination polyhedra are denoted by white vertices, and two vertices are connected by a line if the corresponding polyhedra have a common O atom (Figure 1c). Within the current review, the structures of 24 organically templated uranyl sulfates and selenates are based on the layers of this topology, including compounds **2**–**4**. Being tridentate bridging, sulfate and selenate tetrahedra have their fourth non-shared O atom arranged up or down relative to the plane of the layer. This variability can generate the formation of geometrical isomers with various orientations of tetrahedral groups that can be described by the orientation matrices [90]. Symbols **u** (up), **d** (down), or □ (tetrahedra missing in the graph) are assigned to each tetrahedral site (white vertex) at the graph of the layer (Figure 1c). The aforementioned change in the interlayer space filling results, however, does not entail differences in the geometric isomerism of the layers. Thus, the orientation matrix for the U-bearing layers in the structures of **2**–**4** can be written as (**uud**□)/(□**udd**). Moreover, the degree of layer distortion is also the same. Layer undulation (Figure 2a,b) can be calculated as the shortest interatomic distance between the neighbor wave crests, and the thickness can be calculated as the normal distance between the mean planes that pass through the most protruding parts of the layer (i.e., terminal O atoms of the tetrahedra). The layer undulation and thickness parameters are 7.5 and 5.9 Å, 7.2 and 5.6 Å, 7.4 and 5.9 Å for **2**–**4**, respectively. The substitution of the isopropylammonium cation and H_2_O molecule by a selenous acid molecule and H_3_O ion results in the orthogonalization of the unit cell of **4**, and in the alignment of neighboring layers.

It is known that hydrophilic amine groups of organic cations in the structures of organically templated uranyl compounds prefer to associate with dense fragments of U-bearing substructural complexes (four-membered rings of the graph), while hydrocarbon components of the molecules, which do not play a charge-compensating role, are usually arranged in front of rarefied zones (six-membered rings of the graph). It is of interest that the arrangement of the isopropylammonium cation in the structure of **4** fully corresponds to that in the structures of **2** and **3** (Figure 2c,d). The arrangement of the selenous acid molecule in **4** plays a role of the hydrocarbon part of the second isopropylammonium cation in **2** and **3**, so that the H_3_O^+^ molecule occupies a position different from H_2_O in the structures of **2** and **3**, and functions as an amino group.

## 3. Discussion

### 3.1. Isotypic Uranyl Sulfates and Selenates

An aforementioned example demonstrates the rather high resistance of the U-bearing structural type to substitutions in the oxyanion substructural complex. However, this case in the total amount of known structural data is not so frequent. Only 11 pairs of isotypic sulfate–selenate compounds, excluding those reported here, are known. Most of them account for the uranyl compounds templated by various amino acid molecules (**174**–**185**, **188**, **189** [76]). Two pairs correspond to quite rare piperazine (**122** [47], **123** [48]) and 3-Aminotropane (**154**, **155** [64]) molecules. Additionally, only two pairs of compounds represent more common organic molecules that were used in the synthetic experiments: 1-butylamine (**26** [11], **30** [14]) and tetramethylammonium (**71** [33], **72** [34]). There are also several examples of a very close structural architecture, for example, compounds templated by 1,4-diaminobutane (**47** [12,13], **48** [26]) and N.N-dimethylethylenediamine (**89** [43], **91** [36]). Those pairs of compounds have the same topology of the U-bearing layers, and even close unit cell parameters; however, an arrangement of the respective organic and additional H_2_O molecules in the interlayer space differs, which leads to the impossibility of classifying them as isotypic compounds.

### 3.2. Topology of U-Bearing Complexes

**Figure 3:**
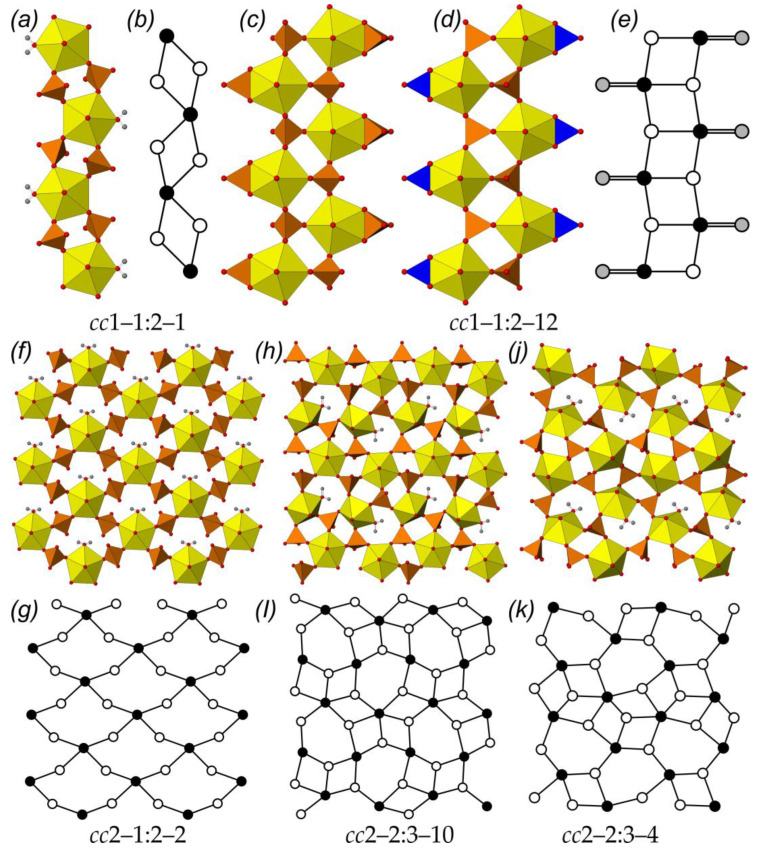
The most common topologies of the U-bearing substructural units among organically templated uranyl sulfate and selenate compounds: chains of *cc*1–1:2–1 (**a**) and *cc*1–1:2–12 (**c**,**d**) types and their black-and-white graphs ((**b**,**e**), respectively); layers of *cc*2–1:2–2 (**f**), *cc*2–2:3–10 (**h**), and *cc*2–2:3–4 (**j**) topologies and their respective graphs (**g**,**i**,**k**). Legend: see Figure 1; blue triangles = NO_3_ groups; gray nodes and double line = edge-shared *T*O_4_ tetrahedra or NO_3_ group.

An analysis of Table 1 demonstrates the following distribution of U-bearing substructural complexes. There are four compounds, of which the structures contain isolated uranyl sulfate or selenate moieties, which possess three different topologies. The crystal structures of 49 compounds are based on the 1D U-bearing chains of 9 various topological types, among which two topologies *cc*1–1:2–12 (13 compounds with [UO_2_(*T*O_4_)_2_]^2–^ or [UO_2_(*T*O_4_)(NO_3_)]^−^ (*T* = S, Se) composition) and *cc*1–1:2–1 (17 compounds with [UO_2_(*T*O_4_)_2_ H_2_O] (*T* = S, Se) composition) account for more than half of all the considered chain-based crystal structures (Figure 3a-e). Compound **96** [42] should be especially mentioned, since it is the only compound within those under consideration, of which the crystal structure is based on units of different topological types (*cc*1–1:1–2 and *cc*1–1:2–8). The vast majority of organically templated uranyl sulfates and selenates (135 compounds) have their structures based on layered U-bearing complexes, which is fully consistent with the general trend for U(VI) compounds [48,91,92,93]. Among them, three topologies that prevail over others can be quite clearly distinguished as well. Those are *cc*2–1:2–2 (16 compounds with [(UO_2_)(*T*O_4_)_2_(H_2_O)]^2–^ (*T* = S, Se) composition), *cc*2–2:3–10 (17 compounds with [(UO_2_)_2_(*T*O_4_)_3_(H_2_O)]^2–^ (*T* = S, Se) composition), and *cc*2–2:3–4 (22 compounds with [(UO_2_)_2_(*T*O_4_)_3_(H_2_O)]^2–^ (*T* = S, Se) composition) (Figure 3f-k). Moreover, the *cc*2–2:3–10 topology of the layered U-bearing complexes was observed in the structures of the compounds templated by 11 various organic molecules; the *cc*2–1:2–2 topology was described for 11 molecules of various shapes and sizes, and the most common topological type, *cc*2–2:3–4, was observed in the structures with 17 various amine molecules. There were five compounds, including compound **1**, of which the structures were based on microporous frameworks. Additionally, the crystal structures of **31**, **166**, and **191** contained nanotubules, formed by vertex-sharing of *Ur* bipyramids and sulfate or selenate tetrahedra. It is of interest that nanotubules in all three compounds can be unfolded into the planar fragments of the *cc*2–3:5–2 topological type.

### 3.3. Structural Complexity

The method of calculating and analyzing structural complexity parameters has been quite successfully used in the study of mineral associations [94,95,96,97], as well as in the analysis of various groups of inorganic compounds, including uranyl compounds [98,99,100,101].

**Figure 4:**
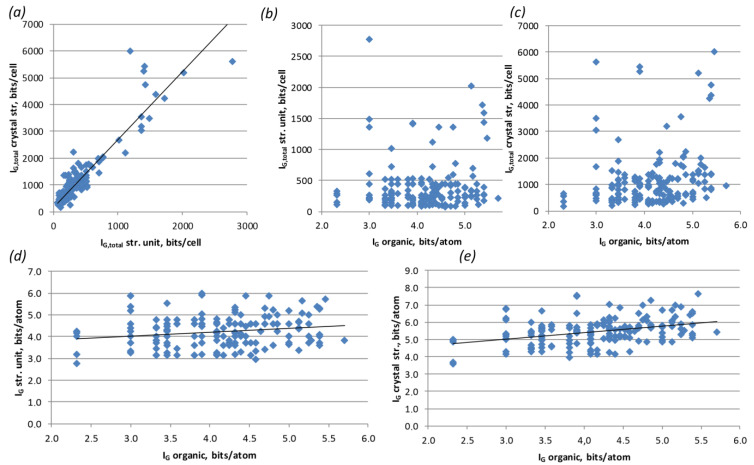
Correlation graphs of structural complexity parameters: complexity of U-bearing structural unit vs. complexity of the entire structure (**a**); complexity of organic molecule vs. complexity of U-bearing structural unit and of the entire structure per unit cell (**b,c**) and per atom (**d**,**e**).

Considering the full set of available structural data, the only obvious correlation was observed between complexities of the U-bearing structural unit and entire structure (Figure 4a).

On the one hand, this trend is rather obvious: the more complex the structural unit, the more complex the structure is. However, one should keep in mind that the most accurate comparison and analysis of the calculated complexity values are possible for compounds with similar chemical compositions (polymorph modifications). Deviations in the chemical composition or, to be more precise, in a number of atoms in the crystal structure automatically create certain allowances, since the complexity parameters directly depend on the number and multiplicity of atomic sites. For example, a single H_2_O molecule introduces three atomic sites into the calculation. Therefore, organic molecules should contribute to the overall complexity due to the large number of atomic sites compared to the inorganic substructural unit. However, there is no such tendency observed in the graphs, if complexity values per unit cell are taken into account (Figure 4b,c). The situation becomes somewhat better when using complexity parameters per atom (Figure 4d,e). However, even here, there were no real trends, minor tendencies. This was mainly due to the fact that organic molecules with similar numbers of atoms had completely different functionalities (size, shape, number of amino groups, etc.), which presented different effects on the U-bearing structural complexes. Therefore, it made sense to consider some groups of molecules separately.

**Figure 5:**
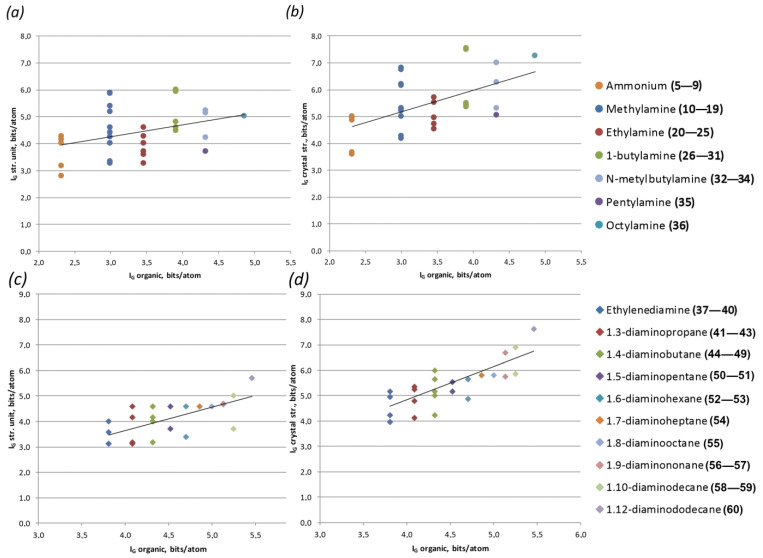
Correlation graphs of chained amine (**a**,**b**) and diamine molecule (**c**,**d**) complexity vs. complexity of U-bearing structural unit (**a**,**c**) and of the entire structure (**b,d**), per atom.

Thus, the most representative groups were the rows of chained amine and diamine molecules. For these groups, firstly, there was a long-term trend towards an increase in the hydrocarbon part of the molecule, and secondly, there were relatively large numbers of representatives to obtain better statistics. Both of these statements are more relevant to the group of diamines; however, in comparison with the other types of molecules, the statistics are, unfortunately, less obvious. As it can be seen from the graph (Figure 5), an increase in the length of the hydrocarbon moiety of the chain amine correlates both with an increase in the complexity of the entire structure (which is expected) and with an increase in the complexity of the uranyl-bearing substructural complex. Of course, the trend line cannot be called absolute, but rather a trend of the average complexity values for each of the molecules.

**Figure 6:**
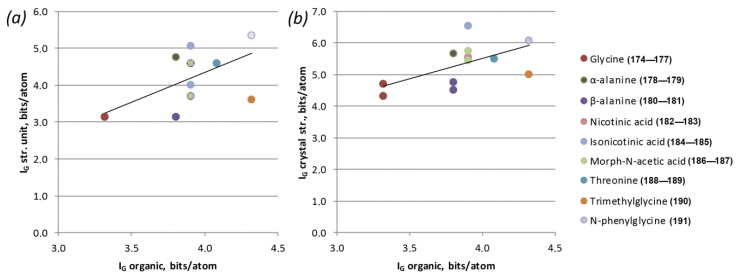
Correlation graphs of amino acid molecule complexity vs. complexity of U-bearing structural unit (**a**) and of the entire structure (**b**), per atom.

A rather good agreement with this tendency can also be observed for compounds with amino acid molecules (Figure 6).

**Figure 7:**
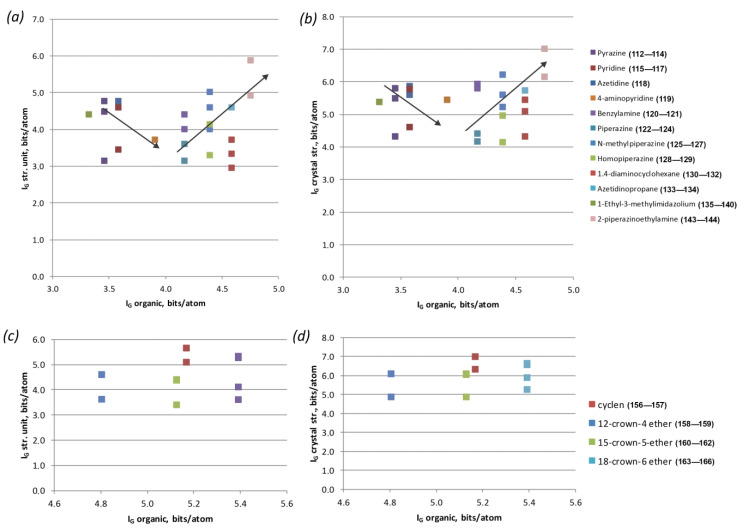
Correlation graphs of cyclic organic molecule complexity vs. complexity of U-bearing structural unit (**a**,**c**) and of the entire structure (**b**,**d**), per atom.

Most of the remaining groups of molecules did not have a large number of compounds available; therefore, it was rather difficult to analyze them. However, several interesting trends could be observed as well. Considering the features of cyclic molecules, one can notice that small strained molecules, such as azetidine, pyridine, imidazole, etc., are located at the beginning of the graph (Figure 7a,b). Those points correspond to rather complex U-bearing structural units, as well as structures in general. As the cycle increases and multiple bonds disappear, the complexity of the substructural building units decrease. Additionally, they begin to increase again as branches from the cyclic base appear.

The importance of the number of atoms is well illustrated in the calculation of complexity parameters by the example of crown molecules (Figure 7c,d). Crown ether molecules do not contain amino groups and are electrically neutral within the structures of the corresponding compounds. Thus, the role of their size in the formation of more complex structures is not clearly traced. This is all the more obvious if one compares the molecules of 12-crown-4 ether and cyclene, which are nearly identical in size and shape. The presence of four amino groups in the structure of the latter, instead of four O atoms, firstly affects the complexity of the molecule itself (eight additional atoms), and secondly increases the complexity of substructural units due to the active participation of amino groups in a particular topology templating process.

## 4. Materials and Methods

### 4.1. Synthesis

Caution: *While isotopically depleted U was used in these experiments, precautions for handling radioactive materials should be followed*.

Uranyl nitrate hexahydrate ((UO_2_)(NO_3_)_2_∙6H_2_O, Vekton, 99%), uranyl acetate ((UO_2_)(CH_3_COO)_2_·2H_2_O, Vekton, 99%), sulfuric acid (H_2_SO_4_, Aldrich, 98%), selenic acid (H_2_SeO_4_, 40 wt. % in H_2_O, Aldrich, 99.95%), 1-butylamine (C_4_H_11_N, Aldrich, ≥99.5%), and isopropylamine (C_3_H_9_N, Aldrich, ≥99.5%) were used as received.

To reveal the features of the isotypic uranyl compounds’ crystallization upon substitution in cationic and anionic substructural complexes, a series of synthetic experiments were conducted. Uranyl sulfate with a microporous structure [C_4_H_12_N]_2_[(UO_2_)_6_(SO_4_)_7_(H_2_O)_2_] (**28**) [11], in the channels of which small-chained molecules of 1-butylamine were arranged, was chosen as the starting point. A similar ratio of initial reagents was taken; however, another small amine with a branched aliphatic part, isopropylamine, was chosen as an organic template.

**Figure 8:**
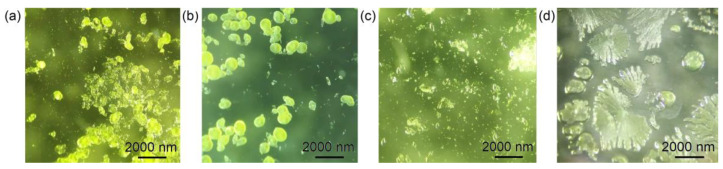
Crystals of **1**–**4** (**a**–**d**, respectively) formed in the described synthetic experiments.

An aqueous solution of 0.1720 g (0.34 mmol) of uranyl nitrate was dissolved in 4 mL of deionized distilled water. Then, 0.500 mL (9.38 mmol) of H_2_SO_4_ and 0.012 mL (0.14 mmol) of isopropylamine were added to the solution, which was stirred until all solid material dissolved. The resulting yellowish transparent solution was left to evaporate in a watch glass at room temperature. Individual, single, flat, rhombic crystals of **1** (Figure 8a) began crystallizing after 3 days. It should be noted that compound **1** was also obtained using another protocol as follows. An aqueous solution of 0.6400 g (1.51 mmol) of uranyl acetate was dissolved in 1 mL of deionized distilled water. Then, 0.200 mL (3.75 mmol) of H_2_SO_4_ (98%) and 0.012 mL (0.14 mmol) of isopropylamine were added to the solution, which was stirred until all solid material dissolved. The resulting yellowish transparent solution was placed in a steel autoclave with a Teflon capsule, which was kept in an oven at a temperature of 180 °C for 24 h. After cooling, the solution was poured onto a watch glass, where individual crystals of **1** began crystallizing after 30 min.

An attempt to crystalize the selenate compound isotypic to **1** was unsuccessful. An analysis of the crystalline precipitate showed that a [C_3_H_10_N]_2_[(UO_2_)_2_(SeO_4_)_3_(H_2_O)](H_2_O) (**2**) phase was formed, which was previously reported in [12,13]. To avoid the accidental crystallization of the compound **2**, several experiments were performed in an extended range of initial reagent concentrations with approximately the same molar ratios. The best-quality single crystals of **2** were formed under the following conditions. An aqueous solution of 0.0880 g (0.18 mmol) of uranyl nitrate was dissolved in 2 mL of deionized distilled water. Then, 0.220 mL (1.79 mmol) of H_2_SeO_4_ (40%) and 0.006 mL (0.07 mmol) of isopropylamine were added to the solution, which was stirred until all solid material dissolved. The resulting yellowish transparent solution was left to evaporate in a watch glass at room temperature. The formation of crystals started in 2 days (Figure 8b). Although the crystal structure of **2** was previously described [12,13], we reported here on the refinement of its structural model with better precision.

To obtain a sulfate compound isotypic to **2**, the following experiment was conducted. An aqueous solution of 0.0880 g (0.18 mmol) of uranyl nitrate was dissolved in 2 mL of deionized distilled water. Then, 0.103 mL (1.92 mmol) of H_2_SO_4_ (98%) and 0.006 mL (0.07 mmol) of isopropylamine were added to the solution, which was stirred until all solid material dissolved. The resulting yellowish transparent solution was left to evaporate in a watch glass at room temperature. The formation of individual, flat, octagonal crystals of **3** started in 3 days (Figure 8c).

The final attempt to substitute isopropylamine in the synthetic protocol of **2** with 1-butylamine molecules was unsuccessful and resulted in the formation of a [C_4_H_12_N][H_3_O][(UO_2_)_2_(SeO_4_)_3_(H_2_O)] (**29**) compound, where the structure was based on the layered complexes with another topology [12,13].

It is of interest that, for the synthesis of **2**, a newly obtained selenic acid was used, while compound **4** was synthesized using a selenic acid reagent stored for ~2 years (Figure 8d). This resulted in the incorporation of electroneutral H_2_SeO_3_ molecules in the interlayer space of **4** (see Chapter 2 for details). The Se(VI) reduction to the 4+ oxidation state during the long-term storage of the selenic acid reagent is a rather frequent process, which was repeatedly noted previously [27,30,100].

### 4.2. Chemical Analysis

The chemical analyses of small pieces of individual single crystals of **1**–**4**, preliminary checked using a single-crystal X-ray diffractometer, were performed using a Hitachi TM 3000 scanning electron microscope equipped with an Oxford EDX spectrometer, with an acquisition time of 30 s per point in an energy dispersive mode (acceleration voltage: 15 kV). The following standards and X-ray lines were used: S—pyrite (FeS_2_), K_α_; Se—PbSe, K_α_; and U—U_3_O_8_, M_β_.

Analytical calculations. Compound **1,** atomic ratio from structural data: U 6.0, S 7.0; found by EDX: U 5.94, S 7.06. Compound **2,** structural data: U 2.0, Se 3.0; found by EDX: U 1.92, Se 3.08. Compound **3,** structural data: U 2.0, S 3.0; found by EDX: U 2.02, S 2.98. Compound **4,** structural data: U 2.0, Se 4.0; found by EDX: U 2.11, S 3.89.

### 4.3. Single-Crystal X-ray Diffraction

Single crystals of **1**–**4** were selected under an optical microscope in polarized light, immersed in an oil-based cryoprotectant, and fixed on cryoloops. Diffraction data were collected at 100 K using a Rigaku XtaLAB Synergy S X-ray diffractometer operated with a monochromated microfocus MoKα PhotonJet-S (λ = 0.71073 Å) source at 50 kV and 1.0 mA, and equipped with a CCD HyPix 6000HE hybrid photon-counting detector [102]. The frame width was 0.5 or 1.0° in ω, and there was a 1 to 16 s count time for each frame. Diffraction data were integrated and corrected for polarization, background, and Lorentz effects using the *CrysAlisPro* program [103]. An empirical absorption correction was applied based on the spherical harmonics (SCALE3 ABSPACK algorithm). The unit-cell parameters (Table 2) were refined using least-squares techniques. The structures were solved by a dual-space algorithm and refined using *SHELX* programs [104,105] incorporated in the *OLEX2* program package [106]. The final models included coordinates and anisotropic displacement parameters for all non-H atoms. The carbon-, nitrogen- and oxygen-bound H atoms were placed in calculated positions and were included in the refinement in the ‘riding’ model approximation, with U*_iso_*(H) set to 1.5U*_eq_*(C) and C–H 0.98 Å for CH_3_ groups, U*_iso_*(H) set to 1.2U*_eq_*(C) and C–H 1.00 Å for tertiary CH groups, U*_iso_*(H) set to 1.2U*_eq_*(N) and N–H 0.91 Å for NH_3_ groups, U*_iso_*(H) set to 1.5U*_eq_*(O) and O–H 0.84 Å for OH^−^ groups, and U*_iso_*(H) set to 1.5U*_eq_*(O) and O–H 0.87 Å for H_2_O molecules. Supplementary crystallographic data for **1**–**4** can be downloaded from the Supplementary Materials section and from the Cambridge Crystallographic Data Centre via www.ccdc.cam.ac.uk/structures/.

### 4.4. Structural Complexity Calculations

A structural complexity approach was recently developed by S.V. Krivovichev [107,108,109,110,111,112]. This method allows estimating the information content of each particular crystal structure, as well as its substructural components. It appears to be quite useful for comparing isotypic or similar structures and quantitatively characterizing the contribution of each substructural component (uranyl sulfate or selenate complexes, interstitial organic template, etc.) to the formation of the whole structural architecture of the compound. The approach is based on the Shannon information content calculations of per atom (*I_G_*) and per unit cell (*I_G,total_*) using the following equations:

(1) $$I_{G}=-\sum_{i=1}^{k}p_{i}\;\log_{2}\;p_{i}\;\;(\mathrm{bits/atom})$$

(2) $$I_{\mathrm{G,total}}=-v\;I_{G}=-v\sum_{i=1}^{k}p_{i}\;\log_{2}\;p_{i}\;(\mathrm{bits/cell})$$

where *k* is the number of different crystallographic orbits (independent sites) in the structure and *p_i_* is the random choice probability for an atom from the *i-*th crystallographic orbit, that is:

*p_i_* = *m_i_*/*v* (3)

where *m_i_* is the multiplicity of the crystallographic orbit (i.e., the number of atoms of a specific Wyckoff site in the reduced unit cell) and *v* is the total number of atoms in the reduced unit cell.

It should be noted that all calculations for already-studied crystal structures were based on the original cif files, which were obtained from structural databases (CCDC and ICSD) and respective publications. In addition, if H-atom sites were not reported in the original entries, they were assigned manually considering the distribution of the H-bonding system. Complexity parameters for the organic molecules and U-bearing substructural complexes were calculated manually, while the parameters for the whole structure were determined using *ToposPro* software [113].

## 5. Conclusions

In this paper, we reviewed the state of the art in the structural chemistry of organically templated uranyl sulfates and selenates, which were considered as the most representative groups of U-bearing synthetic compounds. In total, there were 194 compounds known for both groups, including three novel ones reported here, the crystal structures of which contained 84 various organic molecules. Such statistics illustrates both the great work already performed in the field of syntheses and structural studies, but also the obvious insufficiency of specific system studies, since it turned out that, on average, there were slightly more than two compounds per molecule. Nevertheless, quite clear regularities could be formulated for a number of groups of compounds. Thus, in accordance with the analysis, an increase in the size of the hydrocarbon part and number of charge functional groups of the organic cation led to the formation of rare and more complex topologies.

The presence, albeit in a small number, of isostructural compounds for complex molecules and the absence of such compounds for simpler ones indicated a very fine interaction between the inorganic oxyanion and organic positively charged parts of the structures. Large molecules, apparently, created a kind of a buffer due to their size and the distribution of charge-carrying amino groups, which made it possible to level the difference in the sizes of the sulfate and selenate tetrahedra. However, even in the given examples, the difficulties in obtaining isostructural sulfates and uranyl selenates were very well observed. Thus, compounds **175**, **177**, **179**, and **189** [76] were designated as isostructural, only by the similarity of unit cell parameters, since the quality of the obtained crystals (and all of them were selenates) did not allow one to solve their structures directly. The problem of the presence of a correlation between the uranyl-bearing structural complex topology and the size and shape of the amine molecule has already been raised [12,13,14,64], and it is obvious, at present, that the structural architecture of the entire compound depends on the combination of the organic and oxyanion parts. For example, the most common layer topologies *cc*2–2:3–10, *cc*2–1:2–2, and *cc*2–2:3–4 (see Ch. 3.2) were described in the structures templated by amine molecules of various sizes and shapes (chained, cyclic, etc.); however, the arrangement preserved a certain position of the amino- or other charge-carrying groups. At the same time, changes in the oxyanion substructure can be sometimes realized with symmetry breaking, whilst maintaining the topology of the complex (e.g., **147**, **148** [36]).

This review demonstrated the ability to form isotypic compounds, which, by analogy with recently performed studies in purely inorganic uranyl systems [98,114,115], indicated the probability of the isomorphic sulfate–selenate series’ existence with substitutions in both cationic and oxyanionic moieties. At the same time, the results of the structural studies and topological analysis of all known compounds within the groups under consideration clearly indicate complex crystal chemical limitations in terms of the isomorphic substitution implementation, since the existence of isotypic phases has to date been confirmed only for 24 compounds out of 194, which is slightly above 12%.

## Figures and Tables

**Table 1:** Crystallographic characteristics and structural complexity parameters of organically templated synthetic uranyl sulfates and selenates.

No.	Chemical Formulae	Topology	Sp. Gr.	*a*, Å/α, °	*b*, Å/β, °	*c*, Å/γ, °	Structural Complexity Parameters, Bits per Atom/Bits per Unit Cell			Ref.
							Organic Molecule	U-Bearing Unit	Entire Structure	
	Ammonium, NH_4_^+^			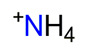			2.322/11.610			
**5**	[NH_4_][(UO_2_)(SO_4_)F]	*cc*2–1:1–7	*Pb*2_1_*a*	8.681(3)/90	11.319(8)/90	6.729(6)/90		3.170/114.117	3.585/172.078	[1]
**6**	[NH_4_]_2_[UO_2_(SO_4_)_2_(H_2_O)](H_2_O)	*cc*2–1:2–2	*P*2_1_/*c*	7.783(5)/90	7.403(2)/102.25(4)	20.918(9)/90		4.250/322.840	4.858/563.526	[2]
**7**	[NH_4_]_4_[(UO_2_)_2_(SO_4_)O_2_)_2_](H_2_O)	5^2^4^3^3^2^	*C*2/*m*	8.6987(15)/90	14.166(2)/104.117(4)	17.847(3)/90		4.150/281.950	4.956/564.949	[3]
**8**	[NH_4_]_2_[(UO_2_)_2_(SO_4_)O_2_)]	5^2^4^3^3^2^	*Cmca*	14.2520(9)/90	8.7748(5)/90	17.1863(10)/90		2.780/144.420	3.654/336.168	[3]
**9**	[NH_4_]_2_[(UO_2_)(SeO_4_)_2_(H_2_O)](H_2_O)_2_	*cc*2–1:2–3	*P*2_1_2_1_2_1_	8.2036(9)/90	11.631(2)/90	14.028(2)/90		4.000/256.000	5.000/640.000	[4]
	Methylamine, CH_3_NH_3_^+^			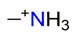			3.000/24.000			
**10**	[CH_6_N]_2_[(UO_2_)_2_(SO_4_)_3_]	*cc*2–2:3–14	*P*1	8.4784(6)/90.170(2)	9.7873(8)/95.744(2)	9.8121(7)/90.136(2)		5.390/226.480	6.209/459.500	[5]
**11**	[CH_6_N][(UO_2_)(SO_4_)(OH)]	6^1^5^2^4^2^3^2^	*Pbca*	11.5951(8)/90	9.2848(6)/90	14.5565(9)/90		3.320/265.750	4.170/600.469	[6]
**12**	[CH_6_N]_2_[(UO_2_)(SeO_4_)_2_ (H_2_O)](H_2_O)	*cc*1–1:2–1	*Pnma*	7.5496(7)/90	12.0135(9)/90	15.8362(13)/90		3.250/208.000	4.272/598.100	[7]
**13**	[CH_6_N]_2_[(UO_2_)(SeO_4_)_2_ (H_2_O)]	*cc*2–1:2–3	*P*2_1_/*c*	8.2366(10) /90	7.5888(6)/104.566(9)	22.260(2)/90		4.000/256.000	5.000/640.000	[7]
**14**	[CH_6_N][H_3_O][(UO_2_)_2_ (SeO_4_)_3_(H_2_O)](H_2_O)	*cc*2–2:3–12	*P*2_1_/*c*	8.4842(10)/90	10.2368(8)/102.803(9)	24.228(2)/90		4.590/440.160	5.285/824.523	[7]
**15**	[CH_6_N]_2_[(UO_2_)_2_(SeO_4_)_3_]	*cc*2–2:3–14	*P*2_1_	8.5827(13)/90	10.0730(15)/95.980(12)	10.0915(14)/90		4.390/184.480	5.209/385.500	[7]
**16**	[CH_6_N]_4_[(UO_2_)_3_(SeO_4_)_5_](H_2_O)_4_	*cc*2–3:5–2	*Pnma*	16.4221(14)/90	18.4773(9)/90	10.3602(5)/90		4.230/608.470	5.311/1657.045	[7]
**17**	[CH_6_N][H_5_O_2_][H_3_O]_2_(UO_2_)_3_(SeO_4_)_5_] (H_2_O)_4_	*cc*2–3:5–2	*Ibca*	20.956(2)/90	34.767(8)/90	18.663(2)/90		5.170/1488.940	6.150/3493.056	[7]
**18**	[CH_6_N]_2_[H_3_O]_2_[(UO_2_)_5_ (SeO_4_)_8_(H_2_O)](H_2_O)_4_	*cc*2–5:8–2	*Pca*2_1_	31.505(2)/90	10.3688(6)/90	16.2424(11)/90		5.860/1359.050	6.807/3049.695	[7]
**19**	[CH_6_N]_1.5_[H_5_O_2_]_1.5_[H_3_O]_3_ [(UO_2_)_5_(SeO_4_)_8_(H_2_O)] (H_2_SeO_4_)_2.6_(H_2_O)_3_	*cc*2–5:8–3	*Pnma*	30.9728(19)/90	37.022(2)/90	10.4171(5)/90		5.880/2776.610	6.749/5614.766	[7]
	Ethylamine, C_2_H_5_NH_3_^+^			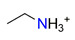			3.459/38.054			
**20**	[C_2_H_8_N][(UO_2_)Cl(SO_4_)(H_2_O)]	*cc*2–1:1–1	*P*2_1_/*c*	8.3545(17)/90	10.550(2)/102.64(3)	12.370(3)/90		3.585/172.078	4.524/416.168	[8]
**21**	[C_2_H_8_N]_2_[(UO_2_)(SeO_4_)_2_(H_2_O)](H_2_O)_2_	*cc*1–1:2–1	*Pnma*	7.6176(9)/90	12.1811(16)/90	19.258(2)/90		3.250/208.000	4.724/944.771	[9]
**22**	[C_2_H_8_N][H_3_O][(UO_2_)(SeO_4_)_2_(H_2_O)]	*cc*1–1:2–1	*P*1	7.5635(15)/79.559(15)	7.6188(15)/89.272(16)	12.101(2)/82.356(16)		4.000/128.000	4.954/307.160	[9]
**23**	[C_2_H_8_N]_3_[(UO_2_)(SeO_4_)_2_(HSeO_4_)]	*cc*1–1:3–2	*P*2_1_/*c*	12.7463(11)/90	12.4261(7)/113.433(6)	14.9928(11)/90		4.248/322.842	5.700/1185.691	[9]
**24**	[C_2_H_8_N][(UO_2_)(SeO_4_)(SeO_2_OH)]	*cc*2–1:2–4	*P*2_1_/*n*	8.475(3)/90	12.264(2)/95.23(3)	10.404(3)/90		3.700/192.423	4.954/614.320	[9]
**25**	[C_2_H_8_N]_2_[(UO_2_)_2_(SeO_4_)_3_(H_2_O)]	*cc*2–2:3–10	*P*2_1_	8.2897(14)/90	12.349(2)/104.439(4)	11.0379(18)/90		4.585/220.078	5.524/508.168	[10]
	1-butylamine, C_4_H_7_NH_3_^+^			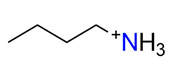			3.907/58.603			
**26**	[C_4_H_10_N]_3_[(UO_2_)_2_(SO_4_)_3_(OH)] (H_2_O)_2_	*cc*2–2:3–10	*P*2_1_	8.439(5)/90	11.912(7)/102.79(10)	10.636(6)/90		4.459/196.215	5.426/466.659	[11]
**27**	[C_4_H_10_N]_8_[(UO_2_)_5_(SO_4_)_9_](H_2_O)	framework	*P*2_1_2_1_2_1_	9.4586(8)/90	26.769(2)/90	32.377(3)/90		5.907/1417.654	7.500/5429.888	[11]
**28**	[C_4_H_10_N]_2_[(UO_2_)_6_(SO_4_)_7_(H_2_O)_2_]	framework	*C*222_1_	10.2776(12)/90	18.339(2)/90	22.788(3)/90		4.800/527.950	5.421/921.596	[11]
**29**	[C_4_H_12_N][H_3_O][(UO_2_)_2_(SeO_4_)_3_(H_2_O)]	*cc*2–2:3–10	*P*2_1_/*c*	10.7691(9)/90	12.5019(12)/98.172(7)	15.4620(14)/90		4.585/440.156	5.492/988.534	[12,13]
**30**	[C_4_H_12_N][H_5_O_2_][(UO_2_)_2_(SeO_4_)_3_(H_2_O)]	*cc*2–2:3–10	*P*2_1_	8.3908(11)/90	12.3602(11)/101.567(10)	10.9150(13)/90		4.459/196.215	5.358/439.319	[14]
**31**	[C_4_H_12_N]_14_[(UO_2_)_10_(SeO_4_)_17_(H_2_O)]	*cc*2–3:5–2 nanotubules	*I*2*mm*	10.8864(5)/90	29.532(2)/90	47.439(2)/90		5.999/1403.665	7.547/5268.064	[15]
	N-methylbutylamine, C_5_H_12_NH_2_^+^			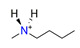			4.322/86.439			
**32**	[C_5_H_14_N]_4_[(UO_2_)_3_(SeO_4_)_4_ (HSeO_3_)(H_2_O)](H_2_SeO_3_)(HSeO_4_)	*cc*2–3:5–3	$P\bar{1}$	11.7068(9)/73.899(6)	14.8165(12)/76.221(7)	16.9766(15)/89.861(6)		5.209/385.500	7.011/1808.897	[16]
**33**	[C_5_H_14_N]_2_[H_3_O][(UO_2_)_3_(SeO_4_)_4_(HSeO_4_) (H_2_O)]	*cc*2–3:5–3	C2/c	16.7572(13)/90	11.7239(12)/98.875(6)	19.0490(13)/90		4.215/295.050	5.306/817.085	[17]
**34**	[C_5_H_14_N]_2_[H_3_O][(UO_2_)_3_ (SeO_4_)_4_(HSeO_4_)(H_2_O)](H_2_O)	*cc*2–3:5–3	*P*2_1_/*n*	10.8252(10)/90	19.0007(10)/100.324(7)	18.6463(15)/90		5.129/718.100	6.267/1930.170	[17]
	Pentylamine, C_5_H_11_NH_3_^+^			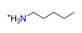			4.322/86.439			
**35**	[C_5_H_14_N][(UO_2_)(SeO_4_)(SeO_2_OH)]	*cc*2–1:2–4	*P*2_1_/*n*	11.553(2)/90	10.6445(16)/108.045(15)	12.138(2)/90		3.700/192.423	5.044/665.860	[18]
	Octylamine, C_8_H_17_NH_3_^+^			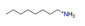			4.858/140.881			
**36**	[C_8_H_20_N]_2_[(UO_2_)(SeO_4_)_2_ (H_2_O)](H_2_O)	*cc*2–1:2–2	$P\bar{1}$	7.498(3)/89.69(3)	11.897(4)/90.05(4)	32.056(14)/88.80(3)		5.000/320.000	7.267/2238.170	[19]
	Ethylenediamine, C_2_H_4_(NH_3_)_2_^2+^			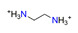			3.807/53.303			
**37**	[C_2_H_10_N_2_][(UO_2_)(SeO_4_)_2_ (H_2_O)](H_2_O)	*cc*2–1:2–2	*C*2/*c*	11.787(2)/90	7.7007(10)/102.016(14)	16.600(3)/90		3.125/100.000	4.225/304.235	[20]
**38**	[C_2_H_10_N_2_][(UO_2_)(SeO_4_)_2_ (H_2_O)](H_2_O)_2_	*cc*2–1:2–2	*P*2_1_/*c*	11.677(2)/90	7.908(1)/98.813(3)	15.698(2)/90		4.000/256.000	5.170/744.469	[10]
**39**	[C_2_H_10_N_2_][(UO_2_)(SeO_3_) (HSeO_3_)](NO_3_)(H_2_O)_0.5_	*cc*2–1:2–4	*Pbca*	13.170(3) /90	11.055(2)/90	18.009(4)/90		3.585/344.156	4.954/1228.641	[21]
**40**	[C_2_H_4_(NH_3_)_2_][UO_2_(SO_4_)_2_H_2_O]	*cc*1–1:2–1	*C*2/*c*	15.6163(4)/90	7.3018(2)/118.731(2)	11.7114(3)/90		3.125/100.000	3.974/238.413	[22]
	1.3-diaminopropane, C_3_H_6_(NH_3_)_2_^2+^			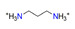			4.087/69.487			
**41**	[C_3_H_12_N_2_][UO_2_(H_2_O)(SO_4_)_2_]	*cc*1–1:2–1	*P*2/*c*	7.2582(2)/90	7.3697(2)/99.4053(19)	11.8514(3)/90		3.125/100.000	4.135/272.930	[23]
**42**	[C_3_H_12_N_2_][(UO_2_)_2_(H_2_O)(SO_4_)_3_]	*cc*2–2:3–4	*P*2_1_/*n*	10.7391(3)/90	10.3791(3)/106.942(1)	18.0265(7)/90		4.585/440.156	5.358/878.639	[23]
**43**	[N_2_C_3_H_12_][UO_2_F(SO_4_)]_2_(H_2_O)	*cc*2–1:1–10	*P*2_1_	6.7745(2)/90	8.1589(2)/94.556(1)	14.3661(4)/90		4.170/150.117	5.248/398.842	[24]
	1.4-diaminobutane, C_4_H_8_(NH_3_)_2_^2+^			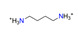			4.322/86.439			
**44**	[C_4_H_14_N_2_]_2_[UO_2_(SO_4_)_3_](H_2_O)_2_	*cc*0–1:3–4	$P\bar{1}$	8.4584(1)/100.8158(5)	10.2830(1)/96.3926(5)	15.2943(2)/112.5170(5)		4.170/150.117	6.000/768.000	[22]
**45**	[C_4_H_14_N_2_][UO_2_(H_2_O)(SO_4_)_2_]	*cc*2–1:2–1	$P\bar{1}$	7.4199(2)/79.1237(9)	7.8380(2)/79.9015(9)	12.0319(3)/83.1098(9)		4.000/128.000	5.170/372.235	[25]
**46**	[C_4_H_14_N_2_][UO_2_F(SO_4_)]_2_	*cc*2–1:1–10	*P*2_1_/c	6.7754(5)/90	8.4094(8)/93.245(3)	14.1492(14)/90		3.170/114.117	4.248/322.842	[25]
**47**	[C_4_H_14_N_2_][(UO_2_)_2_(SeO_4_)_3_(H_2_O)](H_2_O)_2_	*cc*2–2:3–4	*P*2_1_/c	11.068(3)/90	10.455(3)/114.555(19)	20.266(3)/90		4.585/440.156	5.644/1128.771	[12,13]
**48**	(C_4_H_14_N_2_)[(UO_2_)_2_(SO_4_)_3_(H_2_O)]·2H_2_O	*cc*2–2:3–4	*P*2_1_/*n*	10.9075(4)/90	10.4513(4)/97.908(2)	17.7881(7)/90		4.585/440.156	5.644/1128.771	[26]
**49**	[C_4_H_14_N_2_][(UO_2_)(SO_4_)_2_(H_2_O)]·2H_2_O	*cc*2–1:2–3	*P*2_1_/*n*	8.8570(4)/90	7.3299(3)/95.140(2)	20.4260(9)/90		4.000/256.000	5.000/640.000	[26]
	1.5-diaminopentane, C_5_H_10_(NH_3_)_2_^2+^			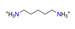			4.524/104.042			
**50**	[C_5_H_16_N_2_][UO_2_(SO_4_)_2_]	*cc*2–1:2–21	*P*2_1_/*c*	7.9825(1)/90	19.8458(4)/111.6563(9)	9.7868(2)/90		3.700/192.423	5.170/744.469	[22]
**51**	[C_5_H_16_N_2_][(UO_2_)_2_(SeO_4_)_3_(H_2_O)]	*cc*2–2:3–10	*P*2_1_	8.0491(11)/90	12.2633(16)/99.918(11)	10.7239(16)/90		4.585/220.078	5.555/522.131	[12,13]
	1.6-diaminohexane, C_6_H_12_(NH_3_)_2_^2+^			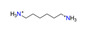			4.700/122.211			
**52**	[C_6_H_18_N_2_][UO_2_(SO_4_)_2_]H_2_O	*cc*1–1:2–12	*P*2_1_/*m*	10.1385(3)/90	6.9537(3)/99.287(2)	11.7233(4)/90		3.393/88.211	4.880/478.242	[22]
**53**	[C_6_H_18_N_2_][(UO_2_)_2_(SeO_4_)_3_(H_2_O)]	*cc*2–2:3–10	*P*2_1_	8.4020(18)/90	12.411(3)/102.951(17)	10.923(2)/90		4.585/220.078	5.644/564.386	[12,13]
	1.7-diaminoheptane, C_7_H_14_(NH_3_)_2_^2+^			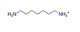			4.858/140.881			
**54**	[C_7_H_20_N_2_][(UO_2_)_2_(SeO_4_)_3_(H_2_O)](H_2_O)	*cc*2–2:3–10	*P*2_1_	8.7100(16)/90	12.4174(14)/101.348(14)	10.8838(18)/90		4.585/220.078	5.807/650.424	[12,13]
	1.8-diaminooctane, C_8_H_16_(NH_3_)_2_^2+^			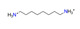			5.000/160.000			
**55**	[C_8_H_22_N_2_][(UO_2_)_2_(SeO_4_)_3_(H_2_O)]	*cc*2–2:3–10	*P*2_1_	8.7793(16)/90	12.4874(15)/100.609(14)	10.9331(18)/90		4.585/220.078	5.807/650.424	[12,13]
	1.9-diaminononane, C_9_H_18_(NH_3_)_2_^2+^			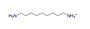			5.129/179.525			
**56**	[C_9_H_24_N_2_][(UO_2_)(SeO_4_)(SeO_2_OH)] (NO_3_)	*cc*2–1:2–4	$P\bar{1}$	10.7480(7) /109.960(1)	13.8847(9)/103.212(2)	14.6363(10)/90.409(1)		4.700/244.423	6.700/1393.691	[27]
**57**	[C_9_H_24_N_2_]_2_[(UO_2_)_3_(SeO_4_)_5_(H_2_O)_2_](H_2_O)x	*cc*2–3:5–4	*P*6_3_/*mmc*	19.5572(5)/90	19.5572(5)/90	47.878(2)/120		4.670/2017.408	5.755/5190.982	[28]
	1.10-diaminodecane, C_10_H_20_(NH_3_)_2_^2+^			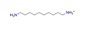			5.248/199.421			
**58**	[C_10_H_26_N_2_][(UO_2_)(SeO_4_)_2_(H_2_O)] (H_2_SeO_4_)_0.85_(H_2_O)_2_	*cc*1–1:2–1	$P\bar{1}$	7.5461(6)/77.678(6)	14.9910(12)/85.463(6)	22.3789(17)/82.717(6)		5.000/320.000	6.895/1640.967	[19]
**59**	[C_10_H_26_N_2_][(UO_2_)(SeO_4_)_2_] (H_2_SeO_4_)_0.5_(H_2_O)	*cc*2–1:2–4	*C*2/*c*	29.280(2)/90	13.3013(10)/93.295(5)	11.4513(7)/90		3.700/192.423	5.879/1375.665	[19]
	1.12-diaminododecane, C_12_H_24_(NH_3_)_2_^2+^			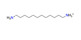			5.459/240.215			
**60**	[C_12_H_30_N_2_]_3_[H_3_O]_2_[(UO_2_)_4_(SeO_4_)_8_] (H_2_O)_5_	*cc*2–1:2–13	*P*2_1_/*n*	11.3437(7)/90	24.8042(12)/96.701(5)	29.2496(19)/90		5.700/1185.691	7.622/6006.177	[29]
	Dimethylamine, C_2_H_6_NH_2_^+^			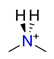			3.459/38.054			
**61**	[C_2_H_8_N]_2_[(UO_2_)(SeO_4_)_2_(H_2_O)]	*cc*1–1:2–1	*P*2_1_2_1_2_1_	7.5363(7)/90	12.2021(11)/90	16.7601(16)/90		4.000/256.000	5.248/797.685	[30]
**62**	[C_2_H_8_N]_2_[(UO_2_)_2_(SeO_4_)_3_(H_2_O)]	*cc*2–2:3–4	*P*2_1_2_1_2_1_	11.2154(5)/90	11.2263(5)/90	16.9138(8)/90		4.585/440.156	5.524/1016.335	[30]
**63**	[C_2_H_8_N]_3_[H_5_O_2_][(UO_2_)_2_(SeO_4_)_3_(H_2_O)_2_]_2_ (H_2_O)_5_	*cc*2–2:3–5	*P*2_1_/*c*	12.451(5)/90	31.126(5)/120.39(2)	14.197(4)/90		5.524/1016.335	6.658/2689.917	[30]
**64**	[C_2_H_8_N]_2_[H_3_O][(UO_2_)_3_ (SeO_4_)_4_(HSeO_3_)(H_2_O)](H_2_SeO_3_)_0.2_	*cc*2–3:5–3	*P*2_1_/*m*	8.3116(4)/90	18.6363(8)/97.582(1)	11.5623(5)/90		4.264/289.947	5.078/619.550	[30]
**65**	[C_2_H_8_N][(H_5_O_2_)(H_2_O)] [(UO_2_)_2_(SeO_4_)_3_(H_2_SeO_3_)](H_2_O)	*cc*2–1:2–14	*P*2_1_/*n*	14.7979(8)/90	10.0238(6)/111.628(1)	16.4176(9)/90		4.755/513.528	5.672/1157.175	[31]
**66**	[C_2_H_8_N]_3_[C_2_H_7_N][(UO_2_)_3_(SeO_4_)_4_ (HSeO_3_)(H_2_O)]	*cc*2–3:5–3	*Pnma*	11.6591(11)/90	14.9556(17)/90	22.194(2)/90		4.472/715.508	5.607/1883.819	[30]
**67**	[C_2_H_8_N]_3_[H_3_O][(UO_2_)_3_(SeO_4_)_4_(SeO_3_) (H_2_O)](H_2_O)	*cc*2–3:5–3	*P*2_1_/*m*	8.941(2)/90	19.300(4)/97.510(4)	11.377(3)/90		4.329/303.050	5.599/996.681	[30]
	Isopropylamine, C_3_H_7_NH_3_^+^			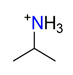			3.807/53.303			
**1**	[C_3_H_10_N]_2_[(UO_2_)_6_(SO_4_)_7_(H_2_O)_2_]	framework	*C*222_1_	10.2560(2)/90	18.4062(4)/90	22.8900(4)/90		4.900/578.152	5.454/949.072	This work
**2**	[C_3_H_10_N]_2_[(UO_2_)_2_(SeO_4_)_3_(H_2_O)](H_2_O)	*cc*2–2:3–4	*P*2_1_/*c*	11.4644(2)/90	11.2426(2)/99.421(2)	18.7555(4)/90		4.585/440.156	5.781/1271.899	This work,[12,13]
**3**	[C_3_H_10_N]_2_[(UO_2_)_2_(SO_4_)_3_(H_2_O)](H_2_O)	*cc*2–2:3–4	*P*2_1_/*c*	11.0470(1)/90	10.8926(1)/100.180(1)	18.5397(2)/90		4.585/440.156	5.781/1271.899	This work
**4**	[C_3_H_10_N](H_3_O)[(UO_2_)_2_ (SeO_4_)_3_(H_2_O)](H_2_SeO_3_)	*cc*2–2:3–4	*P*2_1_/*c*	11.2894(4)/90	11.1012(3)/94.717(3)	18.1368(6)/90		4.585/440.156	5.585/1072.313	This work
	Tert-butylamine, C_4_H_9_NH_3_^+^			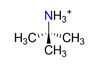			4.087/69.487			
**68**	[C_4_H_12_N]_2_[(UO_2_)(SeO_4_)_2_(H_2_O)]	*cc*2–1:2–3	*C*2/*c*	27.212(10)/90	7.372(3)/117.75(2)	23.113(7)/90		4.000/256.000	5.644/1128.771	[20]
**69**	[C_4_H_12_N]_2_[(UO_2_)_2_(SeO_4_)_3_(H_2_O)]	*cc*2–2:3–4	*P*2_1_/*c*	11.3478(14)/90	11.3850(9)/91.865(11)	18.959(3)/90		4.585/440.156	5.858/1359.052	[12,13]
	Tetramethylammonium, C_4_H_12_N^+^			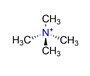			4.087/69.487			
**70**	[C_4_H_12_N][(UO_2_)(SO_4_)(H_2_O)_2_]Cl	*cc*1–1:1–2	*P*2_1_	8.989(6)/90	6.877(4)/109.77(4)	10.981(8)/90		3.807/106.606	5.000/320.000	[32]
**71**	[C_4_H_12_N][(UO_2_)(SO_4_)(NO_3_)]	*cc*1–1:2–12	*C*2/*m*	21.106(1)/90	6.9350(3)/97.5468(18)	8.4284(5)/90		3.252/78.039	4.306/249.763	[33]
**72**	[C_4_H_12_N][(UO_2_)(SeO_4_)(NO_3_)]	*cc*1–1:2–12	*C*2/*m*	21.244(5)/90	7.1092(11)/97.693(17)	8.6581(18)/90		3.252/78.039	4.375/280.000	[34]
**73**	[C_4_H_12_N]_2_[(UO_2_)_6_(SO_4_)_7_(H_2_O)_2_]	framework	*C*222_1_	10.3466(2)/90	18.5415(3)/90	22.7001(4)/90		4.800/527.950	5.487/976.681	[35]
	Triethylamine, C_6_H_15_NH^+^			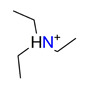			4.524/104.042			
**74**	[C_6_H_16_N][H_3_O][(UO_2_)_2_(SeO_4_)_3_(H_2_O)] (H_2_O)	*cc*2–2:3–4	*P*2_1_	8.8162(16)/90	12.4459(15)/103.695(14)	10.8212(19)/90		4.585/220.078	5.755/621.528	[12,13]
**75**	[C_6_H_16_N][H_5_O_2_][(UO_2_)_2_(SeO_4_)_3_(H_2_O)]	*cc*2–2:3–10	*P*2_1_	8.8477(3)/90	12.4835(5)/103.382(1)	10.8373(4)/90		4.585/220.078	5.755/621.528	[36]
**76**	(H_5_O_2_)[C_6_H_16_N][(UO_2_)_2_(SeO_4_)_3_(H_2_O)]	*cc*2–2:3–10	*P*2_1_/*c*	10.753(1)/90	12.3221(8)/91.050(9)	18.142(2)/90		4.585/440.156	5.755/1243.056	[13]
	Guanidine, CH_6_N_3_^+^			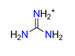			3.322/33.219			
**77**	[CH_6_N_3_]_2_[(UO_2_)(SO_4_)(H_2_O)_2_](NO_3_)_2_ (H_2_O)	*cc*1–1:1–2	*P*2_1_/*n*	12.3824(7)/90	7.0329(4)/99.598(2)	21.5362(12)/90		3.807/213.212	5.492/988.534	[37]
**78**	[CH_6_N_3_]_2_[(UO_2_)(SO_4_)_2_(H_2_O)](H_2_O)_2_	*cc*2–1:2–2	*C*2/*c*	11.220(8)/90	8.027(4)/101.00(7)	18.681(8)/90		3.125/100.000	4.440/372.955	[38]
**79**	[CH_6_N_3_]_2_[(UO_2_)_2_(SO_4_)_3_]	*cc*2–2:3–14	*P*2_1_2_1_2	9.907(3)/90	9.597(3)/90	9.762(3)/90		3.440/144.477	4.480/367.319	[39]
**80**	[CH_6_N_3_]_2_[(UO_2_)(SeO_4_)_2_(H_2_O)](H_2_O)_1.5_	*cc*2–1:2–2	*C*2/*c*	37.314(4)/90	7.1771(6)/109.267(8)	13.2054(14)/90		4.000/256.000	5.352/867.056	[20]
**81**	[CH_6_N_3_]_3_[(UO_2_)_2_(SeO_4_)_3_(HSeO_4_)](H_2_O)_2_	*cc*2–1:2–4	*P*2_1_2_1_2_1_	10.7261(9)/90	13.9178(16)/90	18.3213(17)/90		4.755/513.528	5.977/1506.275	[20]
**82**	[CH_6_N_3_]_2_[(UO_2_)_2_(SeO_4_)_3_]	*cc*2–2:3–14	*P*2	9.9448(15)/90	9.727(2)/90.213(12)	10.1508(15)/90		4.440/186.477	5.480/449.319	[5]
	Aminoguanidine, CH_7_N_4_^+^			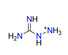			3.585/43.020			
**83**	[CH_7_N_4_]_2_[(UO_2_)(SO_4_)_2_(H_2_O)]	*cc*2–1:2–2	*C*2/*c*	11.297(2)/90	7.8336(16)/100.18(3)	17.984(4)/90		3.125/100.000	4.627/444.156	[40]
	1_,_2-diaminopropane, C_3_H_12_N_2_^2+^			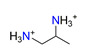			4.087/69.487			
**84**	[C_3_H_12_N_2_]_2_[(UO_2_)_2_(SO_4_)_4_(H_2_O)_4_](H_2_O)_2_	*cc*1–1:2–1	$P\bar{1}$	7.3983(2)/95.1761(12)	7.6333(2)/94.6412(13)	12.5946(5)/96.578(2)		4.248/161.421	5.285/412.261	[41]
**85**	[C_3_H_12_N_2_][UO_2_(H_2_O)(SO_4_)_2_]	*cc*1–1:1–1	$P\bar{1}$	7.3296(2)/92.0309(13)	7.3702(2)/106.041(1)	11.6822(2)/93.6783(9)		4.000/128.000	5.044/332.930	[42]
**86**	[C_3_H_12_N_2_][UO_2_F(SO_4_)]_2_·H_2_O	*cc*2–1:1–9	*Pnma*	13.5775(3)/90	14.6180(4)/90	8.1168(2)/90		3.170/228.235	4.752/912.313	[24]
**87**	[C_3_H_12_N_2_][(UO_2_)(SeO_4_)_2_(H_2_O)_2_](H_2_O)	*cc*0–1:2–3	$P\bar{1}$	7.5611(16)/94.604(18)	7.7650(17)/94.405(17)	12.925(3)/96.470(17)		4.248/161.421	5.285/412.261	[34]
	N.N-dimethylethylene diamine, C_4_H_14_N_2_^2+^			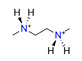			4.322/86.439			
**88**	[C_4_H_14_N_2_][UO_2_(SO_4_)_2_]	*cc*2–1:2–20	*P*2_1_2_1_2_1_	9.3322(1)/90	9.7743(2)/90	13.8897(3)/90		3.700/192.423	5.044/665.860	[43]
**89**	[C_4_H_14_N_2_][(UO_2_)_2_(H_2_O)(SO_4_)_3_](H_2_O)	*cc*2–2:3–4	*P*2_1_/*c*	11.2460(2)/90	10.5387(2)/92.9884(6)	17.0432(3)/90		4.585/440.156	5.555/1044.263	[43]
**90**	[C_4_H_14_N_2_][(UO_2_)(SeO_4_)_2_(H_2_O)]	*cc*2–1:2–8	$P\bar{1}$	6.853(2)/99.62(3)	10.537(3)/94.45(3)	10.574(3)/100.52(3)		4.000/128.000	5.170/372.235	[34]
**91**	[C_4_H_14_N_2_][(UO_2_)_2_(SeO_4_)_3_(H_2_O)](H_2_O)	*cc*2–2:3–4	*P*2_1_/*c*	11.568(4)/90	10.857(4)/95.545(11)	17.229(7)/90		4.585/440.156	5.555/1044.263	[36]
	Diethylenetriamine, C_4_H_15_N_3_^3+^			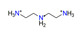			4.459/98.107			
**92**	[C_4_H_15_N_3_][H_3_O]_0.5_[(UO_2_)_2_(SeO_4_)_3_ (H_2_O)](NO_3_)_0.5_	*cc*2–2:3–4	*P*2_1_/*c*	11.1679(4)/98.019(1)	10.9040(4)/90	17.9913(6)/90		4.459/392.430	5.615/1100.483	[30]
	1.3-diaminopentane, C_5_H_16_N_2_^2+^			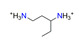			4.524/104.042			
**93**	[C_5_H_16_N_2_]_2_[(UO_2_)(SeO_4_)_2_(H_2_O)](NO_3_)_2_	*cc*1–1:2–1	*C*2/*c*	28.916(5)/90	8.0836(10)/110.909(11)	11.9856(16)/90		3.125/100.000	5.158/722.100	[34]
	N,N-Diethylethylenediamine, C_6_H_18_N_2_^2+^			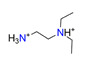			4.700/122.211			
**94**	[C_3_H_8_N]_2_[(UO_2_)_2_(SeO_4_)_3_(H_2_O)](H_2_O)	*cc*2–2:3–4	*P*2_1_/*c*	12.0301(15)/90	10.7845(9)/91.865(10)	17.490(2)/90		4.585/440.156	5.728/1214.319	[13]
	Tetramethylethylenediamine, C_6_H_18_N_2_^2+^			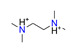			4.700/122.211			
**95**	[C_6_H_18_N_2_][(UO_2_)_2_(SO_4_)_3_(H_2_O)]	*cc*2–2:3–4	*P*2_1_	8.4460(7)/90	11.966(1)/104.043(2)	10.6635(9)/90		4.585/220.078	5.644/564.386	[36]
	1.2-ethylamino ethane*,* C_6_H_18_N_2_^2+^			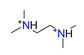			4.700/122.211			
**96**	[C_6_H_18_N_2_][(UO_2_)_2_(H_2_O)_3_(SO_4_)_3_]	*cc*1–1:1–2 *cc*1–1:2–8	$P\bar{1}$	6.8234(1)/101.3691(6)	8.7384(1)/98.1340(6)	19.2381(4)/90.0480(11)		4.907/294.413	5.807/650.424	[42]
	N,N-*diethylethane-1,2-diamine,* C_6_H_18_N_2_^2+^			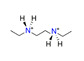			4.700/122.211			
**97**	[C_6_H_18_N_2_]_2_[UO_2_F(SO_4_)]_4_·H_2_O	*cc*2–1:1–6	$P\bar{1}$	10.8832(2)/75.6604(8)	10.9386(2)/73.6101(7)	16.5325(3)/89.7726(7)		5.285/412.261	6.508/1184.419	[24]
	N,N,N′,N′-tetramethyl-1,3-propanediamine, C_7_H_20_N_2_^2+^			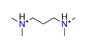			4.858/140.881			
**98**	[C_7_H_20_N_2_][(UO_2_)_2_(SO_4_)_3_(H_2_O)]	*cc*2–2:3–17	$P\bar{1}$	6.7861(1)/88.6230(9)	8.5143(1)/81.6364(8)	19.0442(3)/84.8577(6)		4.585/220.078	5.728/607.160	[44]
	N-(3-aminopropyl)-1,3-propanediamine, N_3_C_6_H_20_^3+^			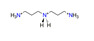			4.858/140.881			
**99**	(N_3_C_6_H_20_)(H_5_O_2_)[(UO_2_)_4_(SO_4_)_6_(H_2_O)_2_]· 4H_2_O	*cc*2–2:3–4	*P*2_1_/*n*	10.8576(1)/90	10.4120(1)/97.518(1)	17.8726(3)/90		4.585/440.156	5.858/1359.052	[45]
**100**	(N_3_C_6_H_20_)[(UO_2_)(SO_4_)_2_(SO_3_OH)]·H_2_O	*cc*1–1:3–2	$P\bar{1}$	7.9164(1)/92.892(1)	11.0632(1)/97.938(1)	11.3354(1)/107.497(1)		4.248/161.421	5.672/578.587	[45]
	Triethylenetetramine, C_6_H_22_N_4_^4+^			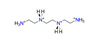			5.000/160.000			
**101**	[C_6_H_22_N_4_][UO_2_(H_2_O)(SO_4_)_2_]_2_(H_2_O)_6_	*cc*1–1:2–8	$P\bar{1}$	6.7186(5)/72.337(2)	9.2625(7)/89.198(2)	13.1078(9)/70.037(1)		4.000/128.000	5.358/439.319	[46]
**102**	[C_6_H_22_N_4_][UO_2_(SO_4_)_2_]_2_	*cc*2–1:2–20	*Pbca*	9.3771(2)/90	12.9523(3)/90	18.9065(6)/90		3.700/384.846	4.858/1127.052	[47]
**103**	[C_6_H_22_N_4_][(UO_2_)(SeO_4_)_2_(H_2_O)](H_2_O)	*cc*2–1:2–3	*P*2_1_/*n*	13.002(2)/90	7.962(1)/114.077(2)	14.754(2)/90		4.000/256.000	5.129/718.100	[10]
**104**	[N_4_C_6_H_22_][UO_2_(H_2_O)(SO_4_)_2_]_2_(H_2_O)_6_	*cc*1–1:2–8	$P\bar{1}$	6.7318(1)/72.3395(6)	9.2975(1)/89.1401(7)	13.1457(3)/70.0267(12)		4.000/128.000	5.358/439.319	[47]
	Tris(2-aminoethyl)-amine, C_6_H_21_N_4_^4+^			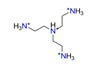			5.000/160.000			
**105**	[C_6_H_21_N_4_][(UO_2_)(SeO_4_)_2_(HSeO_4_)]	*cc*1–1:3–2	*P*2_1_/*m*	9.2218(6)/90	12.2768(9)/116.165(1)	9.4464(7)/90		3.616/137.421	4.931/512.846	[10]
**106**	(N_4_C_6_H_22_)[(UO_2_)_2_(SO_4_)_4_(H_2_O)_2_]·3H_2_O	*cc*2–1:2–2	*P*2_1_/*n*	7.4982(1)/90	16.9531(5)/90.729(2)	11.4496(2)/90		4.000/256.000	5.700/1185.691	[45]
**107**	[C_6_H_22_N_4_]_2_[(UO_2_)_2_(SO_4_)_6_](H_2_O)	*cc*0–1:3–4	$P\bar{1}$	11.2315(1)/88.4073(5)	13.2136(1)/74.5896(5)	14.3521(2)/66.5370(6)		5.170/372.235	6.687/1377.419	[22]
	1_,_5_,_8_,_12-tetraazadodecane, C_8_H_26_N_4_^4+^			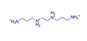			5.248/199.421			
**108**	[C_8_H_26_N_4_][(UO_2_)(SeO_4_)_2_(H_2_O)](H_2_O)	*cc*2–1:2–2	*P*2_1_/*n*	7.8198(11)/90	16.516(3)/90.662(11)	11.6831(16)/90		4.000/256.000	5.285/824.523	[48]
**109**	[C_8_H_26_N_4_]_0.5_[(UO_2_)_2_(SO_4_)_3_(H_2_O)](H_2_O)_2_	*cc*2–2:3–12	*P*2_1_/*n*	11.8400(2)/90	10.3190(2)/107.7718(9)	16.5919(4)/90		4.585/440.156	5.615/1100.483	[49]
	Tetraethylenepentamine, C_8_H_28_N_5_^5+^			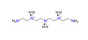			5.358/219.660			
**110**	[C_8_H_28_N_5_]_2_[(UO_2_)_5_(H_2_O)_5_(SO_4_)_10_]H_2_O	*cc*2–1:2–2	*Pbnm*	7.7638(5)/90	14.16890(5)/90	56.46930(5)/90		5.372/1719.017	6.409/4229.773	[47]
	Imidazole, C_3_H_5_N_2_^+^			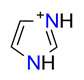			3.322/33.219			
**111**	[C_3_H_5_N_2_]_2_[(UO_2_)_2_(SO_4_)_3_]	*cc*2–2:3–14	*P*2_1_2_1_2_1_	9.7683(3)/90	10.0252(3)/90	19.9136(7)/90		4.392/368.955	5.358/878.639	[42]
	Pyrazine, C_4_H_5_N_2_^2+^			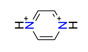			3.459/38.054			
**112**	(C_4_H_5_N_2_)_2_[(UO_2_)(SeO_4_)_2_(H_2_O)]	*cc*2–1:2–1	*C*2/*c*	18.2026(8)/90	7.9997(3)/106.947(2)	11.6866(5)/90		3.125/100.000	4.301/326.842	[50]
**113**	(C_4_H_5_N_2_)_2_[(UO_2_)_2_(SeO_4_)_3_(H_2_O)]·3H_2_O	*cc*2–2:3–11	$P\bar{1}$	8.8130(5)/108.286(2)	11.5642(6)/94.279(2)	13.1308(7)/105.157(2)		4.755/256.764	5.781/635.950	[50]
**114**	(H_3_O)(C_4_H_5_N_2_)_2_[(UO_2_)_3_(SeO_4_)_5_(H_2_O)]· H_2_O	*cc*2–3:5–3	*Pbcm*	11.573(3)/90	19.220(6)/90	14.465(5)/90		4.472/715.508	5.469/1465.712	[50]
	Pyridine, C_5_H_6_N^+^			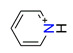			3.585/43.020			
**115 ^1^**	[C_5_H_6_N][(UO_2_)(SeO_4_)(HSeO_3_)]	*cc*2–1:2–4	*P*2_1_/*n*	8.993(3)/90	13.399(5)/108.230(4)	10.640(4)/90		-	-	[51]
**116**	[C_5_H_6_N]_2_[(UO_2_)_2_(SeO_4_)_3_]	*cc*2–2:3–14	*Pccn*	9.987(7)/90	10.251(7)/90	20.957(14)/90		3.440/288.955	4.589/789.318	[52]
**117**	(C_5_H_6_N)_2_[(UO_2_)_2_(SeO_4_)_3_(H_2_O)]∙3H_2_O	*cc*2–2:3–10	*P*2_1_/*n*	10.6354(4)/90	12.3334(5)/103.182(1)	18.8810(8)/90		4.585/440.156	5.755/1243.056	[50]
	Azetidine, C_3_H_8_N^+^			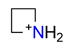			3.585/43.020			
**118**	[C_3_H_8_N]_2_[(UO_2_)_2_(SeO_4_)_3_(H_2_O)]	*cc*2–2:3–4	*P*2_1_2_1_2_1_	10.8620(5)/90	11.1105(5)/90	17.8815(8)/90		4.585/440.156	5.585/1072.313	[53]
	4-aminopyridine, C_5_H_7_N_2_^+^			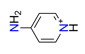			3.907/58.603			
**119**	[C_5_H_7_N_2_]_2_[(UO_2_)(SO_4_)_2_]	*cc*1–1:2–12	$P\bar{1}$	7.0126(9)/68.187(5)	10.3352(13)/78.940(5)	13.8027(19)/71.339(3)		3.700/96.211	5.426/466.659	[37]
	Benzylamine, NC_7_H_10_^+^			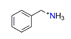			4.170/75.059			
**120**	[NC_7_H_10_]_2_[(UO_2_)_2_(SO_4_)_3_]·H_2_O	*cc*2–2:3–14	*P*2_1_/*n*	10.3238(2)/90	9.1710(2)/91.414(2)	27.1113(7)/90		4.392/368.955	5.907/1417.654	[45]
**121**	[C_7_H_10_N]_2_[(UO_2_)(SeO_4_)_2_(H_2_O)](H_2_O)	*cc*2–1:2–2	*Pna*2_1_	24.221(2)/90	11.9169(11)/90	7.4528(7)/90		4.000/256.000	5.781/1271.899	[10]
	Piperazine, C_4_H_12_N_2_^2+^			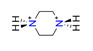			4.170/75.059			
**122**	[C_4_H_12_N_2_][UO_2_(H_2_O)(SO_4_)_2_]	*cc*1–1:2–1	*C*2/*c*	14.7676(3)/90	7.6585(2)/104.837(2)	11.6807(2)/90		3.125/100.000	4.146/281.947	[54]
**123**	[C_4_H_12_N_2_][(UO_2_)(SeO_4_)_2_ (H_2_O)]	*cc*1–1:2–1	*C*2/*c*	15.7651(10)/90	7.4093(5)/101.121(2)	11.9639(8)/90		3.125/100.000	4.146/281.947	[50]
**124**	[C_4_H_12_N_2_]_0.5_[(UO_2_)(HSeO_3_)(SeO_3_)]	*cc*2–1:2–20	*P*2_1_/*c*	10.9378(5)/90	8.6903(4)/90.3040(8)	9.9913(5)/90		3.585/172.078	4.392/368.955	[55]
	1-methylpiperazine, C_5_H_14_N_2_^2+^			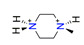			4.392/92.239			
**125**	[C_5_H_14_N_2_][UO_2_(H_2_O)(SO_4_)_2_]	*cc*1–1:2–1	$P\bar{1}$	8.0031(2)/72.704(1)	8.1873(2)/81.7766(11)	10.8911(3)/78.7917(9)		4.000/128.000	5.209/385.500	[56]
	2-methylpiperazine, C_5_H_14_N_2_^2+^			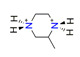			4.392/92.239			
**126**	[C_5_H_14_N_2_][UO_2_(H_2_O)(SO_4_)_2_]	*cc*1–1:2–4	$P\bar{1}$	10.7537(2)/87.998(1)	11.4297(2)/79.660(1)	11.5797(2)/80.6313(6)		5.000/320.000	6.209/918.999	[54]
**127**	[C_5_H_14_N_2_][UO_2_F(H_2_O)(SO_4_)]_2_	*cc*2–1:1–7	*P*2_1_/*n*	8.4354(2)/90	15.5581(4)/96.666(1)	14.8442(6)/90		4.585/440.156	5.585/1072.313	[24]
	Homopiperazine, C_5_H_14_N_2_^2+^			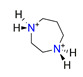			4.392/92.239			
**128**	[C_5_H_14_N_2_]_2_[UO_2_(SO_4_)_3_]	*cc*0–1:3–2	*C*2/*c*	14.4975(3)/90	11.9109(3)/110.475(1)	13.0157(3)/90		3.281/118.117	4.940/592.827	[43]
**129**	[C_5_H_14_N_2_][UO_2_(H_2_O)(SO_4_)_2_]	*cc*1–1:1–2	*P*22_1_2_1_	7.6955(2)/90	11.7717(3)/90	14.7038(4)/90		4.125/264.000	4.125/264.000	[43]
	1.4-diaminocyclohexane, C_6_H_16_N_2_^2+^			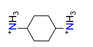			4.585/110.039			
**130**	[N_2_C_6_H_16_][UO_2_F_2_(SO_4_)]	*cc*1–1:1–13	$P\bar{1}$	6.9105(2)/72.659(1)	9.6605(2)/87.068(1)	10.1033(2)/77.957(1)		3.322/66.439	5.087/345.947	[24]
**131**	[C_6_H_16_N_2_][UO_2_F_2_(SO_4_)]	*cc*2–1:1–14	*Pmmn*	6.9503(1)/90	17.2147(4)/90	7.0867(1)/90		2.948/106.117	4.309/534.320	[24]
**132**	[C_6_H_16_N_2_][UO_2_(SO_4_)_2_]·2H_2_O	*cc*1–1:2–12	$P\bar{1}$	6.7813(1)/76.7537(7)	10.0636(2)/75.6074(7)	12.9753(3)/74.3971(13)		3.700/96.211	5.426/466.659	[57]
	Azetidinopropaneamine, C_6_H_16_N_2_^+^			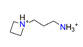			4.585/110.039			
**133**	[C_6_H_16_N_2_][(UO_2_)_2_(SeO_4_)_3_(H_2_O)](H_2_O)	*cc*2–2:3–4	*P*2_1_/*c*	11.3575(5)/90	11.021(5)/90.608(1)	17.8038(8)/90		4.585/440.156	5.728/1214.319	[53]
**134**	[C_3_H_8_N]_2_(H_5_O_2_)[(UO_2_)_2_(SO_4_)_3_(HSO_4_)]	*cc*2–1:2–13	*P*2_1_/*n*	8.677(3)/90	10.294(3)/97.521(7)	26.474(8)/90		4.755/513.528	5.858/1359.052	[53]
	1-ethyl-3-methyl imidazolium, C_6_H_11_N_2_^+^			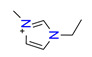			4.248/80.711			
**135 ^1^**	[C_6_H_11_N_2_]_2_[(UO_2_)(SO_4_)_2_]	*cc*1–1:2–12	*C*2/*c*	31.90(1)/90	9.383(5)/93.999(7)	13.770(7)/90		-	-	[58]
**136**	[C_6_N_2_H_11_](Na)[(UO_2_)_4_(SO_4_)_2_(OH)_2_(O)_2_]· 3(H_2_O)	5 ^2^ 4 ^3^ 3 ^2^	*P*2_1_/*c*	17.182(5)/90	8.852(3)/100.693(4)	17.162(5)/90		4.755/513.528	5.803/1288.360	[59]
**137**	[C_6_N_2_H_11_](H_9_O_4_)[(UO_2_)(SO_4_)_2_]	*cc*1–1:2–12	$P\bar{1}$	6.9504(11)/95.993(2)	9.9247(15)/95.024(2)	14.966(2)/103.323(2)		3.700/96.211	5.931/723.550	[59]
**138**	[C_6_N_2_H_11_]_2_[(UO_2_)_2_(SO_4_)_3_(H_2_O)]	*cc*2–2:3–22	$P\bar{1}$	9.5715(11)/81.803(1)	10.4399(12)/81.394(1)	13.7023(16)/86.480(1)		4.585/220.078	5.954/738.320	[59]
**139**	[C_6_N_2_H_11_]_2_[(UO_2_)_2_(SO_4_)_3_(H_2_O)_2_]·2(H_2_O)	*cc*1–2:3–3	*P*2_1_/*n*	12.952(2)/90	19.302(3)/116.891(2)	13.224(2)/90		4.755/513.528	6.150/1746.528	[59]
**140**	[C_6_N_2_H_11_][(UO_2_)_2_(SO_4_)(OH)(O)]	5 ^2^ 4 ^3^ 3 ^2^	$P\bar{1}$	8.859(2)/107.671(3)	8.926(2)/97.350(3)	9.893(3)/104.502(3)		3.807/106.606	5.044/332.930	[59]
	1-(3-aminopropyl) imidazole, N_3_C_6_H_13_^+^			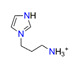			4.459/98.107			
**141**	[N_3_C_6_H_13_][(UO_2_)(SO_4_)_2_]	*cc*1–1:2–12	$P\bar{1}$	6.8164(1)/76.749(1)	7.6357(1)/88.091(1)	14.1979(2)/86.533(1)		3.700/96.211	5.129/359.050	[45]
	1-butyl-3-methylimidazole, C_8_H_15_N_2_^+^			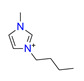			4.644/116.096			
**142**	[C_8_H_15_N_2_]_2_[(UO_2_)_4_(SeO_3_)_5_]	6 ^1^ 5 ^2^ 4 ^2^ 3 ^2^	*Pnma*	18.860(2)/90	18.010(2)/90	11.140(1)/90		4.250/544.000	5.455/1789.277	[52]
	2-piperazinoethylamine, C_6_H_18_N_3_^3+^			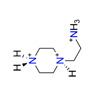			4.755/128.382			
**143**	[C_6_H_18_N_3_][(UO_2_)_2_(H_2_O)(SO_4_)_3_(HSO_4_)] (H_2_O)_4.5_	*cc*2–1:2–12	*P*2_1_/*a*	15.7673(4)/90	10.5813(3)/99.9216(9)	16.7710(5)/90		4.907/588.827	6.129/1716.199	[60]
**144**	[C_6_H_18_N_3_]_2_[(UO_2_)_5_(H_2_O)(SO_4_)_8_](H_2_O)_5_	*cc*2–5:8–2	*P*2_1_/*n*	21.5597(3)/90	10.2901(2)/96.7436(7)	22.8403(3)/90		5.858/1359.052	6.989/3550.252	[60]
	1,4-bis(3-aminopropyl)piperazine, C_10_H_28_N_4_^4+^			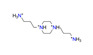			5.392/226.477			
**145**	(N_4_C_10_H_28_)_0.5_[(UO_2_)(SO_4_)_2_(H_2_O)]·H_2_O	*cc*2–1:2–2	*P*2_1_/*n*	7.5484(2)/90	16.9859(4)/90.580(2)	11.4581(3)/90		4.000/256.000	5.322/851.508	[45]
**146**	[C_10_H_28_N_4_][(UO_2_)_2_(SO_4_)_4_]	*cc*2–1:2–20	*Pbca*	9.5831(2)/90	15.6060(3)/90	18.1212(3)/90		3.700/384.846	5.087/1383.790	[61]
	1,2,3-benzotriazole, C_6_H_6_N_3_^+^			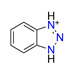			3.907/58.603			
**147**	[C_6_H_6_N_3_][H_5_O_2_][(UO_2_)_2_(SeO_4_)_3_(H_2_O)]	*cc*2–2:3–10	*P*2_1_/*c*	12.167(3)/90	12.316(3)/108.270(4)	14.909(3)/90		4.585/440.156	5.392/905.909	[36]
**148**	[C_6_H_6_N_3_][H_7_O_3_][(UO_2_)_2_(SO_4_)_3_(H_2_O)] (H_2_O)	*cc*2–2:3–10	*C*2	19.678(7)/90	10.600(4)/95.979(7)	10.925(4)/90		4.585/220.078	5.720/594.846	[36]
	Melamine, C_3_H_8_N_6_^2+^			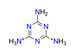			4.087/69.487			
**149**	[C_3_H_8_N_6_][(UO_2_)_2_(SO_4_)_3_(H_2_O)](H_2_O)	*cc*2–2:3–4	*P*2_1_/*n*	11.1194(4)/90	10.5921(3)/101.405(2)	17.0143(6)/90		4.585/440.156	5.459/960.860	[62]
**150**	[(C_3_H_8_N_6_)(SeO_4_)] [(UO_2_)(SeO_4_) (H_2_SeO_3_)_2_]	*cc*2–1:3–6	*P*2_1_/*c*	16.247(4)/90	8.680(2)/90.615(5)	13.347(3)/90		4.644/464.386	5.392/905.909	[63]
	4.4′-Bipyridine, C_10_H_10_N_2_^2+^			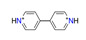			4.459/98.107			
**151**	[C_10_H_10_N_2_][UO_2_(SO_4_)_2_]H_2_O	*cc*1–1:2–12	$P\bar{1}$	6.9507(1)/79.1992(7)	7.7097(1)/80.1403(8)	15.9200(4)/80.9717(14)		3.700/96.211	5.248/398.842	[42]
	Terpyridine, C_15_H_14_N_3_^3+^			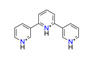			5.000/160.000			
**152**	[C_15_H_14_N_3_][(UO_2_)(SO_4_)_2_](NO_3_)(H_2_O)_2_	*cc*1–1:2–12	$P\bar{1}$	6.9732(7)/111.809(2)	13.569(1)/102.386(2)	13.641(1)/93.833(2)		3.700/96.211	5.781/635.950	[46]
	1.4-diazabicyclo(2.2.2)octane, C_6_H_14_N_2_^2+^			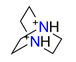			4.459/98.107			
**153**	[C_6_H_14_N_2_][UO_2_(H_2_O)(SO_4_)_2_]	*cc*2–1:2–3	*P*2_1_/*n*	8.6480(1)/90	7.7135(1)/90.7254(9)	21.2554(3)/90		4.000/256.000	5.248/797.685	[54]
	3-Aminotropane, C_8_H_18_N_2_^2+^			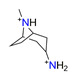			4.807/134.606			
**154**	[C_8_H_18_N_2_](H_5_O_2_)_2_[(UO_2_)_3_(SeO_4_)_5_(H_2_O)] (H_2_O)	*cc*2–3:5–5	*P*2_1_/*n*	10.210(2)/90	19.151(4)/98.959(3)	17.819(3)/90		5.209/770.999	6.340/2054.111	[64]
**155**	[C_8_H_18_N_2_](H_5_O_2_)_2_[(UO_2_)_3_(SO_4_)_5_(H_2_O)] (H_2_O)	*cc*2–3:5–5	*P*2_1_/*n*	10.147(3)/90	18.726(6)/99.043(7)	17.076(5)/90		5.209/770.999	6.322/2023.017	[64]
	Cyclen, C_8_H_24_N_4_^4+^			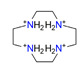			5.170/186.117			
**156**	[C_8_H_24_N_4_][(UO_2_)_3_(SO_4_)_5_] (H_2_O)_3_	*cc*2–3:5–2	*Pna*2_1_	16.8623(10)/90	18.0113(11)/90	10.1928(6)/90		5.087/691.895	6.304/1991.995	[64]
**157**	(C_8_H_24_N_4_)(H_3_O)_2_[(UO_2_)_4_(SeO_4_)_7_(H_2_O)] (H_2_O)_6.75_	*cc*2–4:7–3	$P\bar{1}$	8.7587(14)/73.807(3)	13.067(2)/88.980(4)	23.009(4)/86.129(3)		5.644/564.386	6.977/1758.275	[64]
	12-crown-4 ether, C_8_H_16_O_4_			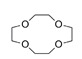			4.807/134.606			
**158**	[C_8_H_16_O_4_]_0.5_[UO_2_(SO_4_)(H_2_O)](H_2_O)	*cc*1–1:1–2	$P\bar{1}$	7.007(1)/91.31(1)	8.0408(6)/93.60(2)	10.776(2)/100.18(1)		3.585/86.039	4.858/281.763	[65]
**159**	[C_8_H_16_O_4_]_2_[(H_5_O_2_)_3_(H_9_O_4_)] [(UO_2_)_2_(SeO_4_)_3_(H_2_O)]_2_	*cc*2–2:3–10	*P*2_1_/*c*	10.7328(6)/90	12.2828(5)/110.102(5)	22.7085(17)/90		4.585/440.156	6.087/1655.790	[66,67]
	15-crown-5-ether, C_10_H_20_O_5_			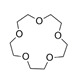			5.129/179.525			
**160**	[K@(C_10_H_20_O_5_)][(UO_2_)(SeO_4_)(HSeO_4_) (H_2_O)]	*cc*1–1:2–1	*Pnma*	15.386(3)/90	10.771(2)/90	13.239(3)/90		3.382/229.947	4.860/1030.319	[68]
**161**	[(H_5_O_2_)(H_3_O)_3_](C_10_H_20_O_5_)[(UO_2_)_3_ (SeO_4_)_5_(H_2_O)]	*cc*2–3:5–3	*P*2_1_/*m*	11.6754(5)/90	18.9887(10)/112.282(3)	12.2047(5)/90		4.399/325.500	6.064/1491.859	[66,67]
**162**	[(H_5_O_2_)x(H_3_O)_4_-_x_](C_10_H_20_O_5_) [(UO_2_)_3_(SeO_4_)_5_(H_2_O)](H_2_O)_y_	*cc*2–3:5–3	*C*2/*c*	24.2575(15)/90	11.7501(7)/101.996(1)	18.9243(12)/90		4.362/340.261	6.012/1527.126	[66,67]
	18-crown-6 ether, C_12_H_24_O_6_			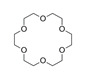			5.392/226.477			
**163**	[C_12_H_24_O_6_]_0.5_[(UO_2_)(SO_4_)(H_2_O)_3_]	*cc*1–1:1–1	*P*2_1_/*n*	9.314(5)/90	9.339(3)/103.62(3)	16.734(3)/90		4.087/277.947	5.248/797.685	[65]
**164**	[(H_3_O)@(C_12_H_24_O_6_)]_2_(H_3_O)_8_ [(UO_2_)_14_(SO_4_)_19_(H_2_O)_4_](H_2_O)_20.5_	framework	*I4*/*m*	28.023(1)/90	28.023(1)/90	19.6840(7)/90		5.313/1583.312	6.531/4375.972	[69]
**165**	[K@(C_12_H_24_O_6_)][(UO_2_)(SeO_4_)(NO_3_)] (H_2_O)	*cc*1–1:2–12	*P*2_1_/*c*	7.2402(2)/90	21.2024(7)/91.581(1)	15.7322(5)/90		3.585/172.078	5.858/1359.052	[70]
**166**	[(H_3_O)@(C_12_H_24_O_6_)]K[H_3_O]_2_ [(UO_2_)_3_(SeO_4_)_5_](H_2_O)_4_	*cc*2–3:5–2 nanotubules	*Ccmm*	11.292(1)/90	37.158(1)/90	38.504(1)/90		5.264/1431.790	6.622/4754.269	[69]
	Benzo-15-crown-5 ether, C_14_H_20_O_5_			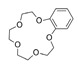			5.285/206.131			
**167**	[C_14_H_20_O_5_]_0.5_[(UO_2_)(SO_4_)(H_2_O)_2_](H_2_O)	*cc*1–1:1–2	$P\bar{1}$	6.908(2)/79.46(2)	8.717(4)/75.28(2)	13.578(2)/89.98(3)		3.807/106.606	5.524/508.168	[65]
	Thiourea, CN_2_H_4_S			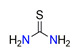			3.000/24.000			
**168**	[CN_2_H_4_S]_2_[UO_2_(SO_4_)_2_]·0.3H_2_O	*cc*1–1:2–12	*P*2_1_2_1_2_1_	6.9283(1)/90	13.3983(3)/90	15.2250(3)/90		3.700/192.423	5.044/665.860	[71]
	Chloroacetamide, ClCH_2_CONH_2_			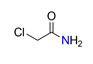			3.322/33.219			
**169**	(C_2_H_4_NCOCl)[UO_2_(SO_4_)(H_2_O)_2_]	*cc*1–1:1–2	$P\bar{1}$	6.892(3)/104.40(3)	8.786(6)/109.71(3)	9.494(6)/90.33(3)		3.807/106.606	4.524/208.084	[72]
	Choline, C_5_H_12_NO^+^			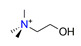			4.248/80.711			
**170**	[C_5_H_12_NO][(UO_2_)(SeO_4_)Cl(H_2_O)]	*cc*2–1:1–1	*P*2_1_/*n*	10.745(4)/90	11.236(4)/114.580(5)	12.477(4)/90		3.585/172.078	5.044/665.860	[73]
	3-hydroxypiperidine, C_5_H_7_NO^+^			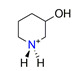			3.807/53.303			
**171**	[(C_5_H_7_NO)_2_(H_2_O)][(UO_2_)_2_(SeO_4_)_3_ (H_2_O)_2_](H_2_O)	*cc*2–2:3–11	$P\bar{1}$	9.4248(7)/85.456(1)	11.2711(8)/79.571(1)	13.1059(10)/73.439(1)		4.585/220.078	5.781/635.950	[36]
	Carbamoylguanidine, C_2_N_4_H_7_O_2_^2+^			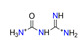			3.907/58.603			
**172**	[C_2_N_4_H_7_O][(UO_2_)(SO_4_)(OH)](H_2_O)_0.5_	6 ^1^ 5 ^2^ 4 ^2^ 3 ^2^	*P*2_1_/*c*	10.5135(7)/90	11.3744(7)/110.880(2)	9.2731(5)/90		3.170/114.117	4.747/503.160	[74]
	1-(hydroxyethyl)-5-nitroimidazole (Metronidazole), C_6_H_10_N_3_O_3_^+^			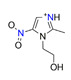			4.459/98.107			
**173**	[(C_6_H_10_N_3_O_3_)(H_5_O_2_)_2_(H_2_O)][(H_5_O_2_)_3_ (H_2_O)][(UO_2_)_5_(SO_4_)_8_(H_2_O)]	*cc*2–5:8–2	*P*2/*c*	18.1693(17)/90	10.0732(10)/103.427(2)	30.098(3)/90		5.858/1359.052	6.858/3182.103	[75]
	Glycine, C₂H₅NO₂^+^			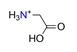			3.322/33.219			
**174**	[(glyH_2_^+^)(H_2_O)]_2_[(UO_2_)(SO_4_)_2_(H_2_O)]	*cc*2–1:2–2	*C*2/*c*	11.5914(5)/90	7.3412(3)/103.993(2)	23.5958(9)/90		3.125/100.000	4.684/468.386	[76]
**175 ^2^**	[(glyH^+^)(H_2_O)]_2_[(UO_2_)(SeO_4_)_2_(H_2_O)]	*cc*2–1:2–2	*C*2/*c*	11.5854(5)/90	7.3322(3)/103.623(2)	23.5768(9)/90		3.125/100.000	4.684/468.386	[76]
**176**	(glyH^+^)_2_[(UO_2_)(SeO_4_)_2_(H_2_O)	*cc*2–1:2–2	*P*2/*c*	7.646(2)/90	9.496(3)/104.832(6)	11.477(3)/90		3.125/100.000	4.301/326.842	[76]
**177 ^3^**	(glyH^+^)_2_[(UO_2_)(SO_4_)_2_(H_2_O)]	*cc*2–1:2–2	*P*2/*c*	7.690(2)/90	9.505(3)/104.805(6)	11.433(3)/90		3.125/100.000	4.301/326.842	[76]
	α-alanine, C_3_H_8_NO_2_^+^			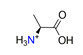			3.807/53.303			
**178**	(α-AlaH^+^)(H_5_O_2_)(H_2_O)_3_[(UO_2_)_2_(SO_4_)_3_ (H_2_O)_2_]	*cc*2–2:3–5	*P*2_1_/*c*	11.000(2)/90	15.402(3)/91.320(6)	13.688(3)/90		4.755/513.528	5.644/1128.771	[76]
**179 ^4^**	(α-AlaH^+^)(H_5_O_2_)(H_2_O)_3_[(UO_2_)_2_(SeO_4_)_3_ (H_2_O)_2_]	*cc*2–2:3–5	*P*2_1_/*c*	11.150(3)/90	15.510(2)/92.00(2)	13.500(5)/90		4.755/513.528	5.644/1128.771	[76]
	β-alanine, C_3_H_8_NO_2_^+^			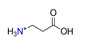			3.807/53.303			
**180**	(β-AlaH^+^)_2_[(UO_2_)(SO_4_)_2_(H_2_O)]	*cc*1–1:2–1	*C*2/*c*	20.660(3)/90	7.3138(11)/91.934(5)	11.8449(17)/90		3.125/100.000	4.739/492.846	[76]
**181**	(β-AlaH^+^)_2_[(UO_2_)(SeO_4_)_2_(H_2_O)]	*cc*1–1:2–1	*C*2/*c*	20.909(2)/90	7.4754(8)/92.589(2)	12.1693(13)/90		3.125/100.000	4.505/396.430	[76]
	Nicotinic acid, C_6_H_6_NO_2_^+^			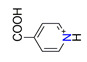			3.907/58.603			
**182**	[(nicH^+^)(H_5_O_2_)(H_2_O)][(UO_2_)(SO_4_)_2_ (H_2_O)]	*cc*2–2:3–10	*P*2_1_/*n*	12.4322(9)/90	11.9693(9)/106.574(2)	14.5768(11)/90		4.585/440.156	5.487/976.681	[76]
**183**	[(nicH^+^)(H_5_O_2_)(H_2_O)][(UO_2_)(SeO_4_)_2_ (H_2_O)]	*cc*2–2:3–10	*P*2_1_/*n*	12.616(2)/90	12.329(3)/107.221(5)	14.819(3)/90		4.585/440.156	5.550/1032.284	[76]
	Isonicotinic acid, C_6_H_6_NO_2_^+^			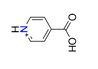			3.907/58.603			
**184**	(IsonicH^+^)_2_[(UO_2_)(SO_4_)_2_(H_2_O)]	*cc*1–1:2–1	$P\bar{1}$	8.5774(9)/97.034(2)	11.2800(12)/105.214(2)	11.4608(12)/106.737(2)		4.000/128.000	5.524/508.168	[76]
**185**	(IsonicH^+^)_2_[(UO_2_)(SeO_4_)_2_(H_2_O)]	*cc*1–1:2–1	$P\bar{1}$	8.629(2)/98.22(5)	11.588(3)/105.180(4)	11.588(3)/105.180(4)		5.044/166.465	6.524/600.168	[76]
	Protonated morpholino-N-acetic acid, C_6_H_6_O_3_^+^			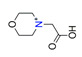			3.907/58.603			
**186**	Na(C_6_H_6_O_3_)[(UO_2_)_2_(SeO_4_)_3_(H_2_O)](H_2_O)_2_	*cc*2–2:3–10	*P*2_1_/*c*	10.7767(5)/90	12.2679(5)/92.126(1)	17.9043(8)/90		4.585/440.156	5.728/1214.319	[77]
**187**	Na_2_(SO_3_OH)(C_6_H_6_O_3_)[(UO_2_)(SO_4_)_2_]	*cc*1–1:2–12	$P\bar{1}$	6.860(3)/85.186(6)	10.546(4)/88.017(5)	13.047(5)/79.752(5)		3.700/96.211	5.426/466.659	[77]
	Threonine, C_4_H_9_NO_3_^+^			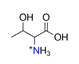			4.087/69.487			
**188**	[(TrhH^+^)(H_2_O)]_2_[(UO_2_)_2_(SO_4_)_3_(H_2_O)]	*cc*2–2:3–4	*P*2_1_2_1_2_1_	10.5155(6)/90	10.516(1)/90	17.3804(12)/90		4.585/440.156	5.492/988.534	[76]
**189 ^5^**	[(TrhH^+^)(H_2_O)]_2_[(UO_2_)_2_(SeO_4_)_3_(H_2_O)]	*cc*2–2:3–4	*P*2_1_2_1_2_1_	10.5602(6)/90	10.485(5)/90	17.5804(2)/90		4.585/440.156	5.492/988.534	[76]
	Trimethylglycine, C_5_H_12_NO_2_^+^			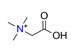			4.322/86.439			
**190**	[C_5_H_12_NO_2_][UO_2_(Cl)(SO_4_)(H_2_O)]	*cc*2–1:1–1	*P*2_1_/*n*	9.0486(7)/90	12.5735(9)/111.4560(7)	12.3064(9)/90		3.585/172.078	5.000/640.000	[78]
	Protonated N-phenylglycine, C_8_H_9_NO_2_^+^			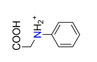			4.322/86.439			
**191**	Na(C_6_H_5_CH(NH_2_)CO_2_)_7_[(UO_2_)_6_(SO_4_)_10_] (H_2_O)_3.5_	*cc*2–3:5–2 nanotubules	*R3m*	44.001(10)/90	44.001(10)/90	10.367(2)/90		5.329/1119.149	6.062/2218.650	[79]
	1-methyl-3-carboxy methylimidazolium, C_6_H_10_N_2_O_2_^+^			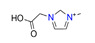			4.322/86.439			
**192**	(C_7_H_15_N_2_O_2_)(H_3_O)[(UO_2_)_2_(SO_4_)_3_(H_2_O)]· 1.5H_2_O	*cc*2–2:3–4	*P*2_1_/*n*	10.7858(6)/90	10.7092(6)/98.493(1)	19.776(1)/90		4.585/440.156	5.755/1243.056	[80]
	N-(3-aminopropyl)-2-pyrrolidinone, C_7_H_14_N_2_O^+^			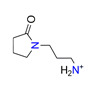			4.585/110.039			
**193**	(N_2_C_6_H_17_COOH)[(UO_2_)_2_(SO_4_)_3_(H_2_O)]	*cc*2–2:3–4	*P*2_1_/*c*	11.4656(3)/90	10.6562(2)/99.604(3)	17.7267(5)/90		4.585/440.156	5.728/1214.319	[45]
	N,N′-bis(3,5-dicarboxylatophenyl)-4,4′-bipyridinium dihydrate, C_26_H_16_N_2_O_8_^2+^			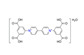			5.700/296.423			
**194**	(C_26_H_16_N_2_O_8_)_0.5_[(UO_2_)(SO_4_)(H_2_O)_2_]·H_2_O	*cc*1–1:1–2	*C*2/*c*	6.8993(14)/90	18.396(4)/93.191(7)	27.847(5)/90		3.807/213.212	5.426/933.318	[81]

^1^—Structural data not available; ^2^—assumed to be the structural analog of **174**; ^3^—assumed to be the structural analog of **176**; ^4^—assumed to be the structural analog of **178**; ^5^—assumed to be the structural analog of **188**.

**Table 2:** Crystallographic data and refinement parameters for **1**–**4**.

Compound	1	2	3	4
*Crystallographic Data*				
Space Group	*C*222_1_	*P*2_1_/*c*	*P*2_1_/*c*	*P*2_1_/*c*
*a* [Å]	10.2560(2)	11.4644(2)	11.0470(1)	11.2894(4)
*b* [Å]	18.4062(4)	11.24259(17)	10.8926(1)	11.1012(3)
*c* [Å]	22.8900(4)	18.7555(4)	18.5397(2)	18.1368(6)
*β* [°]	90	99.421(2)	100.180(1)	94.717(3)
*V* [Å^3^]	4321.03(15)	2384.77(8)	2195.77(4)	2265.30(12)
*Z*	4	4	4	4
*Data Collection Parameters*				
Angle range 2*θ* [^o^]	6.94–55.00	7.12–52.00	6.49–55.00	6.65–55.00
Total reflections	21,967	28,650	71,790	18,562
Unique reflections	4968	4656	5027	5195
Reflections with *F*^2^ > 2σ(*F*^2^)	4715	4326	4773	4616
*R*_int_, *R*_σ_ [%]	4.19, 3.63	4.14, 2.93	7.86, 2.72	2.77, 2.93
*Refinement Parameters*				
*R*_1_ (*F*^2^ > 2σ(*F*^2^)), *wR*_2_ (*F*^2^ > 2σ(*F*^2^)) [%]	2.88, 6.61	2.29, 4.99	1.86, 4.48	2.44, 4.69
*R*_1_ and *wR*_2_ (all data) [%]	3.12, 6.69	2.61, 5.09	2.02, 4.53	3.11, 4.86
S	1.052	1.068	1.048	1.024
ρ_max_, ρ_min_ [e^−^ Å^−3^]	2.008/−1.932	1.940/−1.026	1.477/−1.733	1.453/−0.883
CCDC	2,285,071	2,285,072	2,285,073	2,285,074

## Data Availability

Not applicable.

## Supplementary Materials

The following supporting information can be downloaded at: https://www.mdpi.com/article/10.3390/ijms241613020/s1.
